# Impact of Prescription Drug Monitoring Programs on Postpartum Opioid Prescribing Practices

**DOI:** 10.1111/1475-6773.70123

**Published:** 2026-05-04

**Authors:** Sai Vijay Trilochan Reddy Tadi, CeCe Cheng, Alexander Testa, John J. Byrne, Carolina Vivas‐Valencia

**Affiliations:** ^1^ Department of Biomedical Engineering and Chemical Engineering University of Texas at San Antonio San Antonio Texas USA; ^2^ Department of Obstetrics and Gynecology, Division of Maternal‐Fetal Medicine University of Washington Seattle WA USA; ^3^ Department of Management, Policy and Community Health, School of Public Health University of Texas Health Science Center at Houston San Antonio Texas USA; ^4^ Department of Obstetrics and Gynecology, Division of Maternal‐Fetal Medicine University of Texas Health Science Center at San Antonio San Antonio Texas USA

**Keywords:** opioid prescription, PDMPs, postpartum, state mandates

## Abstract

**Objective:**

To evaluate the association between Prescription Drug Monitoring Programs (PDMPs) and postpartum opioid prescribing from 2007 to 2015, prior to the 2016 Centers for Disease Control and Prevention opioid prescribing guidelines, and to assess differences between low‐ and high‐risk populations.

**Study Setting and Design:**

This cross‐sectional study captures delivery episodes across the United States, primarily from commercially insured patients. The exposure was the implementation of PDMPs at the state level from 2007 to 2015, with states classified by whether implementation occurred before or after 2016. We used a difference‐in‐difference design for analysis. The primary outcome included: (1) morphine milligram equivalents (MME) per day; (2) pills dispensed; (3) average days' supply, and (4) total number of prescriptions filled during the postpartum period (42 days after delivery).

**Data Sources and Analytic Sample:**

We analyzed secondary data from IQVIA PharMetrics, an administrative claims database from 2006 to 2015. We identified reproductive‐age patients (ages 12–59) with a documented delivery and stratified them into low and high‐risk groups by postpartum prescribing and pre‐delivery opioid exposure.

**Principal Findings:**

Among 361,165 delivery episodes (9.9% cesarean, 90.1% vaginal), PDMP implementation was not associated with changes in postpartum opioid prescribing. In an age‐and comorbidity‐adjusted analysis by risk groups, PDMP implementation was not associated with statistically significant changes in MME per day per delivery in either low‐risk (−0.35; 95% CI, −3.27 to 2.57) or high‐risk groups (1.11; 95% CI, −7.46 to 9.68) in the implementation year, and results for pills dispensed, days' supply, and total prescriptions were similarly null, with confidence intervals spanning zero.

**Conclusion:**

State‐level PDMP implementation was not associated with changes in postpartum opioid prescribing. These findings suggest PDMPs alone may be insufficient to influence postpartum prescribing, highlighting the need for alternative strategies to optimize pain management and reduce opioid‐related risks.

## Summary

- What is known on this topic
  ○ Opioid prescriptions are frequently issued following childbirth, particularly after cesarean delivery (CD).
  ○ Prescription Drug Monitoring Programs (PDMPs) are statewide electronic databases tracking controlled substances prescriptions and dispensation, serving as foundations for state‐level efforts to reduce inappropriate opioid use.
  ○ Most PDMP evaluations have focused on the general adult population or people living with chronic pain, leaving a critical gap in understanding the impact of PDMPs in the postpartum period.
- What this study found
  ○ State‐wide implementation of PDMPs before federal guidelines did not have a significant impact on immediate postpartum opioid prescribing patterns across states.
  ○ More targeted interventions for the postpartum population are necessary to improve pain management and reduce the risks associated with opioid use.

## 1 Introduction

The postpartum period is a crucial time when effective pain management must be carefully balanced with the risks of opioid use. Opioid prescriptions are frequently issued following childbirth, particularly after cesarean delivery (CD), which accounts for 32.3% of all deliveries in 2023 [1]. Over half of patients filled an opioid prescription within a week of delivery [2]. While opioids can help manage acute pain during recovery, prolonged or high‐dose use can increase the risk of misuse, opioid use disorder (OUD), and overdose [3, 4, 5]. Insufficient pain control may result in functional impairment, delayed recovery, and, in some instances, a shift to illicit substance use [6, 7, 8]. Research shows that postpartum patients without prior exposure to opioids who filled a postpartum opioid prescription after delivery are at higher risk for serious opioid‐related events compared to those who did not have a prescription [9]. These risks prompted federal and state strategies to improve opioid prescribing practices [10, 11].

Prescription Drug Monitoring Programs (PDMPs) are statewide electronic databases tracking controlled substances prescriptions and dispensation, serving as foundations for state‐level efforts to reduce inappropriate opioid use [10]. The earliest documented PDMP in the United States dates to 1914, with New York State's short‐lived system under the Boylan Act, followed by California's program in 1939; both relied on paper‐based duplicate prescription forms mailed to state databases [10]. Modern electronic PDMPs began with Hawaii's implementation in 1982, and internet connectivity in the 1990s enabled real‐time querying, spurring rapid expansion through the 2000s until all states had operational PDMPs [10, 12, 13]. PDMPs collect information on pill quantity, days' supply, and opioid potency, enabling clinicians to review a patient's controlled medication history before issuing new prescriptions [13]. Furthermore, the Centers for Disease Control and Prevention (CDC) opioid prescribing guidelines (CDC guideline), first published in 2016, recommend prescribing the lowest effective opioid dose and limiting the duration of therapy for acute pain, typically 3 days or less, with more than 7 days rarely necessary. The guidelines also note that opioid dosages at or above 90 morphine milligram equivalents (MME) per day are associated with a significantly increased risk of overdose and serious harm [14].

Early evaluations of PDMPs suggest potential reductions in opioid prescribing [15, 16, 17, 18, 19, 20], though subsequent research has found limited or inconsistent effects when programs were not mandated, poorly integrated into clinical workflows, or were introduced alongside other opioid‐related policies [21, 22, 23]. Most PDMP evaluations have focused on the general adult population or people living with chronic pain, leaving a critical gap in understanding the impact of PDMPs in the postpartum period [24, 25, 26]. The postpartum period has unique clinical needs, substantial variability in prescribing practices, and increased risks of both under‐ and over‐treatment of pain [27]. To address this gap, we utilized a multi‐year administrative claims dataset that includes prescription records from the postpartum population across the 50 states and the District of Columbia (DC). This study investigates the early expansion period from 2007 to 2015 to evaluate the impact of PDMPs prior to the release of major federal guidelines, such as the 2016 CDC guideline, and to determine whether these effects vary between higher‐risk and lower‐risk pregnancy episodes.

## 2 Methods

### 2.1 Study Data and Population

In this cross‐sectional study, we used the IQVIA PharMetrics Plus for Academics Closed Health Plan claims database, which includes a large, geographically diverse sample of the United States population. It includes annual medical and pharmacy claims, such as National Drug Code (NDC) information, quantity dispensed, days supplied, and strength for each prescription. Diagnoses and procedures are identified using the International Classification of Diseases (ICD) and Current Procedural Terminology (CPT) codes. Demographic variables include age, sex, state, and insurance type.

We identified reproductive‐aged patients 12 to 59 years who experienced at least one pregnancy episode between 2006 and 2015, using delivery codes from ICD‐9 and ICD‐10. The deliveries included vaginal delivery (VD) or cesarean delivery (CD), including singleton and multiple‐fetal gestation births. When the diagnosis code did not specify the delivery type, VD was assumed [28, 29]. We identified any opioid prescriptions before and after each pregnancy episode. A complete list of ICD‐9 and ICD‐10 codes and opioid analgesics is available in the Supporting Information: Materials (see Tables S1–S4).

A pregnancy episode was defined as having both a recorded pregnancy diagnosis date and a delivery date. We included pregnancy episodes with continuous enrollment and with available claims for at least 90 days prior to delivery and 42 days following delivery (see Figure S1). Opioid prescriptions were identified using NDC codes from the pharmacy claims. To evaluate postpartum opioid prescribing patterns following the implementation of PDMPs, all eligible pregnancy episodes were retained regardless of whether an opioid prescription was filled in the postpartum period or if there was opioid exposure before delivery. Among delivery episodes with an opioid prescription, we excluded prescription claims with missing, zero, negative, or extremely large (i.e., 999) day‐supply values, as well as those dispensing more than 200 pills, or non‐oral formulations, such as injectables and bulk powder formulations for compounding [30, 31]. Episodes with missing state of residence were also excluded (see Figure S2).

### 2.2 Multiple Pregnancy Episodes

We analyzed data related to pregnancy episodes rather than patient‐level data, as each pregnancy and postpartum period can differ significantly among women. We defined each pregnancy as a separate episode if there were at least 210 days between the delivery date of the first pregnancy and the diagnosis date of the subsequent pregnancy, corresponding to a minimum of 24 weeks of gestation plus a 6‐week postpartum period (see Figure S3) [32, 33].

### 2.3 Risk Stratification

Risk stratification was based on opioid prescribing patterns during the 42‐day period following the delivery, as well as opioid exposure prior to delivery [34]. Opioid‐naïve was defined as no opioid exposure within 90 days prior to delivery [35]. Low‐risk episodes were defined as pregnancy cases in which patients were opioid‐naïve before delivery and received no more than two opioid prescriptions with a total days' supply of five or fewer within the 42‐day postpartum [36]. High‐risk episodes were defined as pregnancy episodes meeting any one of the following criteria: not opioid‐naïve prior to delivery, had a documented history of OUD, or received three or more opioid prescriptions with a total days' supply exceeding 5 days or a cumulative opioid dose exceeding 700 MME during the 42‐day postpartum period [36]. OUD was identified using ICD‐9 and ICD‐10 codes recorded 90 days prior to delivery [37]. A complete list of ICD‐9 and ICD‐10 codes used to identify OUD is provided in the supplemental materials (see Table S5).

### 2.4 Exposure and Outcomes

We defined the establishment of a PDMP as the year in which a state's PDMP system became operational and accessible to prescribers electronically. Beginning in 2016, CDC guidelines for opioid prescribing were adopted, coinciding with widespread implementation of state‐level opioid control policies, including mandatory PDMP use, dosage limits, and prescribing restrictions [13, 14]. We included states with PDMPs established before 2016 to assess their impact before the 2016 CDC guidelines. This cutoff allowed evaluation of PDMPs' impact in a policy environment not yet influenced by national guidelines. We excluded 12 states that had implemented PDMPs before 2006 or in 2006, as they lacked baseline trends. Using this criterion, our final sample consisted of 36 states that implemented PDMPs between January 2007 and December 2015. Pennsylvania, Missouri, and DC had not yet established PDMPs by 2016 (see Supplemental Table S6).

Outcomes of interest included (1) total MME per day, (2) total quantity of opioid pills dispensed, (3) average day's supply of opioid pills, and (4) total number of prescriptions filled during the postpartum period. All outcomes were aggregated at the pregnancy episode level. The postpartum period was defined as the 42 days following delivery. We excluded pregnancy episodes with non‐palliative prescriptions with MME per day greater than 90, as prescribing above this threshold is uncommon for postpartum patients [38].

### 2.5 Total Morphine Milligram Equivalents (MME) Per Day

For each postpartum opioid prescription, we calculated the corresponding MME by multiplying the quantity dispensed, the strength per unit, and a CDC MME conversion factor, then dividing by the days' supply [14]. The total MME per day for each pregnancy episode was calculated by summing these values across all postpartum opioid prescriptions.

### 2.6 Total Quantity of Opioid Pills Dispensed

The total number of opioid pills dispensed was calculated by summing the quantities from all opioid prescriptions filled during the postpartum period for each patient.

### 2.7 Average Day's Supply of Opioid Pills

The average day's supply was calculated as the mean day's supply across all opioid prescriptions filled during the postpartum period for each pregnancy episode.

### 2.8 Total Number of Prescriptions Filled

The total number of prescriptions was determined by summing all opioid claims filled during the postpartum period for each pregnancy episode. This measure reflects both the occurrence and frequency of opioid prescriptions within the defined postpartum window.

### 2.9 Statistical Analysis

We conducted a difference‐in‐differences analysis to compare postpartum opioid prescribing before and after PDMPs implementation, contrasting states that had already adopted them with those that had not prior to 2016. The unit of analysis was the pregnancy episode, with each pregnancy observed once and assigned to a delivery year. PDMP exposure was assigned at the state–year level. Pregnancy episodes delivered in the year that the PDMP was implemented or later were considered exposed, while those in earlier years served as controls. By comparing states that had established PDMPs to states that had not yet established PDMPs within the same calendar years and relative to pre‐treatment outcomes, this design accounts for time‐invariant state characteristics and common temporal shocks. The postpartum outcomes were measured within a fixed window following delivery and attributed to the delivery year for analytic consistency. Episodes occurring late in calendar year could have postpartum claims extending into subsequent year, however all outcomes were attributed to the delivery year to preserve alignment between exposure timing and analytic year assignment. Additional details on the DID specifications, data construction, and model implementation are provided in the supplemental materials (see Supporting Information: Methods).

Traditional two‐way fixed effects (TWFE) estimators can be problematic in a staggered adoption setting, where states implement policies in different years. TWFE assumes that untreated states provide a reasonable counterfactual for treated states, but when policy implementation is staggered across states, as with the adoption of PDMPs, this assumption can yield biased results. This occurs because the duration of post‐policy periods varies across units, and the composition of treated and untreated groups changes over time [39]. Additionally, TWFE models use already treated units as controls for newly treated units, which can distort average treatment effects (ATT) estimates [39, 40]. To address this, we used a two‐step approach combining a stacked DID with the Callaway and Sant'Anna (C&S) estimator [41].

First, we constructed a balanced model, known as the stacked DID, where all treatment states contribute data from 2 years before and 3 years after PDMP implementation, relative to implementation [42]. Second, we applied the C&S estimator to this stacked model [43]. This estimated ATT by comparing each treated cohort to never‐treated or not‐yet‐treated control groups, avoiding the use of already treated units as controls, and allowing for heterogeneous treatment effects across cohorts and time periods.

Finally, we implemented the doubly robust (DR) estimator, which combines outcome regression with inverse probability weighting [44]. This method provides unbiased estimates when either the outcome model or propensity score model is correctly specified [29, 44]. We used repeated cross‐sectional data at the pregnancy episode level, adjusting for maternal age at delivery and selected medical and mental health comorbidities, with standard errors clustered at the state level. Maternal age was included given its association with postoperative opioid prescribing and underlying health conditions. Selected comorbidities (e.g., asthma, hypertension, diabetes, depression) were also included as covariates; corresponding ICD‐9 and ICD‐10 codes are provided in the supplemental materials (see Table S7) [45]. Control states were those that had not yet implemented PDMPs before 2016. The analysis was conducted using the “did” Package developed by Callaway and Sant’ Anna in R version 4.3.1. We considered statistical significance at *p* value < 0.05.

### 2.10 Sensitivity Analysis

We conducted sensitivity analyses to evaluate the robustness of our findings. First, we ran an unadjusted model to assess the impact of adjusting maternal age and medical and mental health comorbidities on the results. Second, we performed subgroup analyses by delivery type (CD vs. VD) to examine whether PDMP implementation effects vary by delivery type. Third, we re‐estimated the model without excluding prescriptions with MME per day exceeding 90.

The University of Texas at San Antonio Institutional Review Board exempted this study from review because it did not involve direct interaction with participants. We followed the STROBE reporting guidelines.

## 3 Results

Between 2006 and 2015, we identified 361,165 pregnancy episodes, with 35,918 (9.9%) resulting in CD and 325,247 (90.1%) being VD (see Table 1). Within 42 days postpartum, 41.5% of CD patients and 17.4% of VD patients filled at least one opioid prescription. On average, cesarean delivery patients were older (mean age CD: 31.74 years vs. VD: 30.15 years) and more likely to be classified as high risk (17.0% vs. 7.0%). CD patients showed higher opioid exposure: they received more MME per day (22.51 vs. 8.92), a greater number of pills (15.67 vs. 5.85), a longer average days' supply (2.53 vs. 0.97 days), and more prescriptions during the postpartum period (0.49 vs. 0.20). Most patients in both groups were aged 25–34 years, but a higher percentage of CD patients were 35 years or older (33.2% vs. 23.6%). Private insurance was the most common payer (77.5% vs. 77.2%). CD had higher rates of comorbidities such as hypertension (8.9% vs. 5.3%), diabetes (15.0% vs. 10.4%), asthma (1.8% vs. 1.6%), and depression (2.4% vs. 2.2%), while OUD and palliative care prevalence were low in both groups.

**TABLE 1:** Demographic Characteristics, Payment Type, Year of Delivery, Covariates, and Risk Groups with Average MME per Day, Pills Dispensed, Days' Supply, and Prescriptions, Stratified by Delivery Type.

Characteristic	Delivery type	
	Cesarean	Vaginal
	*n* (%)	*n* (%)
Number of Patients	35,918	325,247
Year of delivery		
2006	2397 (6.7)	20,376 (6.3)
2007	4020 (11.2)	35,349 (10.9)
2008	4760 (13.3)	39,916 (12.3)
2009	4611 (12.8)	38,471 (11.8)
2010	3403 (9.5)	33,054 (10.2)
2011	3635 (10.1)	32,866 (10.1)
2012	3336 (9.3)	29,169 (9.0)
2013	3169 (8.8)	29,274 (9.0)
2014	3228 (9.0)	32,370 (10.0)
2015	3359 (9.4)	34,402 (10.6)
Maternal age (SD)	31.74 (5.87)	30.15 (5.90)
Age group		
Younger than 25	4186 (11.7)	57,940 (17.8)
25–34	19,809 (55.2)	190,404 (58.5)
35 and Older	11,923 (33.2)	76,903 (23.6)
Primigravida	26,599 (74.1)	233,430 (71.8)
Region		
Midwest	8357 (23.3)	88,786 (27.3)
Northeast	12,789 (35.6)	88,355 (27.2)
South	4978 (13.9)	61,569 (18.9)
West	9794 (27.3)	86,537 (26.6)
Insurance type		
Private Insurance	27,837 (77.5)	251,210 (77.2)
Medicaid	6064 (16.9)	57,356 (17.6)
Cash	2017 (5.6)	16,681 (5.1)
Postpartum opioid prescription (Any)^a^	14,923 (41.5)	56,638 (17.4)
Risk Groups		
Low	29,824 (83.0)	302,607 (93.0)
High	6094 (17.0)	22,640 (7.0)
Medical and mental health covariates^b^		
Asthma	642 (1.8)	5176 (1.6)
Hypertension	3202 (8.9)	17,295 (5.3)
Depression	852 (2.4)	7264 (2.2)
Diabetes	5370 (15.0)	33,709 (10.4)
OUD	69 (0.2)	508 (0.2)
Palliative	1 (0.0)	9 (0.0)
Mean MME per day (SD)	22.51 (32.53)	8.92 (22.82)
Mean pills dispensed (SD)	15.67 (23.39)	5.85 (15.81)
Mean days' supply (SD)	2.53 (4.67)	0.97 (3.05)
Mean number of opioid prescriptions (SD)	0.49(0.66)	0.20(0.48)

Abbreviations: MME, morphine milligram equivalents; OUD, opioid use disorder; SD, standard deviation.

^a^ Postpartum opioid prescription defined as at least one or more filled oral opioid prescriptions within the postpartum observation window following delivery. Opioid prescriptions were identified using national drug codes from pharmacy claims within 42 days following delivery and linked to the pregnancy episode.

^b^ Medical and mental health covariates that were reported for each patient.

### 3.1 DID Estimates

In covariate‐adjusted DID analyses stratified by risk status, state PDMP implementation was not statistically significantly associated with any opioid prescribing outcomes examined (see Tables 2 and 3; see Tables S8 and S9).

**TABLE 2:** Difference‐in‐differences estimates of low‐risk pregnancy episodes postpartum opioid prescribing before and after PDMP implementation.

Year pre/post law implementation	ATT	95% CI
Total MME per day		
−1	0.65	−1.63 to 2.94
0	−0.35	−3.27 to 2.57
1	0.70	−2.14 to 3.54
Total pill dispensation		
−1	0.21	−0.53 to 0.96
0	−0.20	−1.79 to 1.39
1	0.23	−1.26 to 1.71
Average days' supply		
−1	0.02	−0.08 to 0.12
0	−0.03	−0.22 to 0.17
1	0.02	−0.21 to 0.25
Total number of prescriptions filled		
−1	0.01	−0.02 to 0.04
0	−0.01	−0.06 to 0.05
1	0.01	−0.05 to 0.06

*Note:* Values represent covariate adjusted DID estimates using the Callaway and Sant'Anna doubly robust estimator. Models adjust for age at delivery and medical and mental health comorbidities, including asthma, hypertension, diabetes, and depression. Estimates are shown for 1 year before PDMP implementation (−1), the year of implementation (0), and 1 year after implementation (1). All outcomes were measured within the 42‐day postpartum period (0–42 days following delivery) and aggregated at the pregnancy‐episode level.

Abbreviations: ATT, average treatment effect; CI, confidence interval; DID, difference‐in‐differences; MME, morphine milligram equivalents; PDMP, prescription drug monitoring program.

**TABLE 3:** Difference‐in‐differences estimates of high‐risk pregnancy episodes postpartum opioid prescribing before and after PDMP implementation.

Year pre/post law implementation	ATT	95% CI
Total MME per day		
−1	−2.09	−12.17 to 7.99
0	1.11	−7.46 to 9.68
1	1.66	−15.59 to 18.91
Total pill dispensation		
−1	−2.71	−10.89 to 5.47
0	0.71	−6.84 to 8.26
1	0.53	−20.6 to 21.67
Average days' Supply		
−1	−0.55	−2.52 to 1.42
0	0.26	−1.82 to 2.33
1	0.33	−3.89 to 4.55
Total number of prescriptions filled		
−1	−0.07	−0.3 to 0.16
0	0.03	−0.25 to 0.32
1	0.02	−0.39 to 0.43

*Note:* Values represent covariate adjusted DID estimates using the Callaway and Sant'Anna doubly robust estimator. Models adjust for age at delivery and medical and mental health comorbidities, including asthma, hypertension, diabetes, and depression. Estimates are shown for 1 year before PDMP implementation (−1), the year of implementation (0), and 1 year after implementation (1). All outcomes were measured within the 42‐day postpartum period (0–42 days following delivery) and aggregated at the pregnancy‐episode level.

Abbreviations: ATT, average treatment effect; CI, confidence interval; DID, difference‐in‐differences; MME, morphine milligram equivalents; PDMP, prescription drug monitoring program.

#### 3.1.1 Low‐Risk Pregnancy Episodes

Among the low‐risk population, PDMP implementation was not associated with statistically significant changes in opioid prescribing outcomes. For total MME per day, we observed a small, nonsignificant increase of 0.65 (95% confidence interval [CI], −1.63 to 2.94) in the year before implementation (event time = −1). This was followed by a nonsignificant decrease of −0.35 (95% CI, −3.27 to 2.57) in the implementation year (event time = 0), and a modest increase of 0.70 (95% CI, −2.14 to 3.54) 1 year after implementation (event time = 1). None of these were statistically significant.

For total pill dispensation, the number of pills increased slightly in the year before implementation (0.21; 95% CI, −0.53 to 0.96), declined during the implementation year (−0.20; 95% CI, −1.79 to 1.39), and increased modestly 1 year after implementation (0.23; 95% CI, −1.26 to 1.71). These changes were not statistically significant.

For the average days' supply, estimates were near null across all event times, including a small increase in the year before implementation (0.02; 95% CI, −0.08 to 0.12), a slight decrease during the implementation year (−0.03; 95% CI, −0.22 to 0.17), and a small increase 1 year after implementation (0.02; 95% CI, −0.21 to 0.25), none of which were statistically significant.

For the total number of prescriptions filled, estimates were near null across all event times. We observed a small, nonsignificant increase of 0.01 prescriptions (95% CI, −0.02 to 0.04) in the year before implementation (event time = −1), followed by a slight decrease during the implementation year (−0.01; 95% CI, −0.06 to 0.05). An 1 year after implementation, the estimated effect was 0.01 prescriptions per delivery (95% CI, −0.05 to 0.06). None of these differences was statistically significant.

#### 3.1.2 High‐Risk Pregnancy Episodes

Among the high‐risk population, PDMP implementation was not statistically significantly associated with opioid prescribing outcomes. For total MME per day, we observed a nonsignificant decrease of −2.09 (95% confidence interval [CI], −12.17 to 7.99) in the year before implementation (event time = −1), followed by a nonsignificant increase of 1.11 (95% CI, −7.46 to 9.68) in the implementation year (event time = 0), and an increase of 1.66 (95% CI, −15.59 to 18.91) 1 year after implementation (event time = 1). Confidence intervals were wide at all events, and none of these differences were statistically significant.

For total pill dispensation, estimates suggested a nonsignificant decline in the year before implementation (−2.71; 95% CI, −10.89 to 5.47), followed by small, nonsignificant increases during the implementation year (0.71; 95% CI, −6.84 to 8.26) and 1 year after implementation (0.53; 95% CI, −20.60 to 21.67).

For the average day's supply, we observed a nonsignificant decrease of −0.55 days (95% CI, −2.52 to 1.42) in the year before implementation, followed by nonsignificant increases during the implementation year (0.26; 95% CI, −1.82 to 2.33) and one‐year post‐implementation (0.33; 95% CI, −3.89 to 4.55).

For the total number of prescriptions filled, PDMP implementation was not associated with statistically significant changes at any event time. We observed a small, nonsignificant decrease of −0.07 prescriptions (95% CI, −0.30 to 0.16) in the year before implementation (event time = −1), followed by a modest increase during the implementation year (0.03; 95% CI, −0.25 to 0.32). A 1 year after implementation, the estimated effect was 0.02 prescriptions (95% CI, −0.39 to 0.43). None of which were statistically significant.

### 3.2 Sensitivity Analysis

#### 3.2.1 Low‐Risk Pregnancy Episode

Sensitivity analyses yielded results consistent with the primary findings. Estimates from the unadjusted models were similar to those from the covariate‐adjusted specifications across all outcomes and event times, with no statistically significant associations observed. Re‐estimating models without excluding prescriptions with MME per day greater than 90 did not change results. For total MME per day, estimated effects remained small and nonsignificant across all event times (event time = −1: 0.65; 95% CI, −1.45 to 2.76; event time = 0: −0.35; 95% CI, −3.06 to 2.36; event time = 1: 0.70; 95% CI, −2.01 to 3.40), with comparable null patterns for total pill dispensation, average days' supply, and prescriptions filled during postpartum period. Subgroup analyses by delivery type showed no significant associations during the event time = 0. The results for MME per day were as follows: for VD −0.36; 95% CI, −3.51 to 2.78, and for CD, −0.24; 95% CI, −21.18 to 20.71. Comparable null patterns were observed for pills dispensed, days' supply, and prescriptions filled. Additional results are available in Tables S10–S13.

#### 3.2.2 High‐Risk Pregnancy Episode

Sensitivity analyses among the high‐risk population yielded results consistent with the primary findings. Estimates from the unadjusted models and models including prescriptions with MME per day greater than 90 were similar to the covariate‐adjusted results, with no statistically significant associations observed across any outcome or event time. The estimated effects for MME were small and nonsignificant throughout all event times (event time = −1: −2.22; 95% CI, −33.65 to 29.20; event time = 0: 2.50; 95% CI, −17.75 to 22.74; event time = 1: 5.36; 95% CI, −20.28 to 30.93). Similar nonsignificant patterns were observed for total opioid pills dispensed, average days' supply, and total number of opioid prescriptions filled at all event times. Subgroup analyses stratified by delivery type also demonstrated null associations. Among high‐risk VD, PDMP implementation was not associated with statistically significant changes in any prescribing outcomes during the implementation year, including total MME per day (event time = 0: 0.63; 95% CI, −9.57 to 10.84). Similarly, among high‐risk CD, effect estimates during the implementation year were imprecise with wide confidence intervals and did not demonstrate statistically significant changes for any prescribing outcome, including total MME per day (2.50; 95% CI, −112.27 to 117.26). Additional results are available in Tables S14–S17.

## 4 Discussion

Our study demonstrates that state‐wide PDMPs implementation before 2016 did not have a significant impact on immediate postpartum opioid prescribing patterns across states, regardless of risk status. PDMP implementation showed no significant effect on the likelihood of receiving any opioid prescription. These findings were consistently demonstrated even when adjusting for age at delivery and medical and mental health comorbidities. Across all sensitivity analyses, estimated treatment effects were small and not statistically significant. These results suggest that the limited impact of PDMPs implementation on postpartum opioid prescribing patterns is demonstrated across model specifications and delivery subgroups.

The limited effectiveness of PDMPs in the postpartum setting may reflect several factors. It is important to note that our study period (2007–2015) corresponds to a period of PDMP adoption, during which implementation timing varied across states. Additionally, it is essential to distinguish between PDMPs and prescription mandates. While PDMPs primarily function as a monitoring tool that tracks controlled substance prescriptions, a state may have an operational PDMP without a mandate requiring providers to check it [46]. Even with PDMP use mandates, providers may be required to enroll and check PDMPs prior to prescribing opioids, but do not have to adhere to specific limits for the MMEs, number of pills, or number of days prescription patients can receive [47]. The aim of PDMPs is to uncover high‐risk prescribing patterns, while actual prescribing patterns are left to the discretion of the provider. Opioid prescribing limits or state prescribing cap laws that limit the dose or duration of opioid prescriptions did not become more widely implemented until after 2016, and varied by state [29, 41, 48]. Even the implementation of policies targeting prescribing limits did not show a statistically significant difference in prescribing patterns for the postpartum population [29, 49]. During our study period, PDMP consultation was voluntary in most states, and clinicians primarily consulted PDMPs when specific red flags were present, such as early refill requests, reports of lost medications or requests for specific opioids by name [50]. These red flag behaviors may be less characteristic of routine acute post‐surgical prescribing compared to chronic pain management. In the immediate postpartum period, where opioid prescribing typically occurs as part of standardized discharge, providers may be less likely to identify patterns warranting PDMP consultation. Broader clinical guidelines like the 2016 CDC guidelines were associated with accelerated declines in opioid prescribing across multiple measures, as they established MME thresholds, including MME per day, days' supply, and high‐dose prescribing (≥ 90 MME per day) [51, 52].

Previous studies on the association of PDMP implementation with opioid prescribing patterns have shown mixed findings. In a 2018 nationally, representative cross‐sectional survey of physicians evaluating the impact of PDMPs in patients with non‐cancer chronic pain, no link was found between physicians' reports of pain medication or opioid prescribing and the use of PDMPs [53]. However, in their multivariable analysis, those authors did demonstrate that patient characteristics such as non‐white race/ethnicity and Medicaid coverage were associated with a higher likelihood of physicians prescribing opioids [53]. These differences may reflect the social, racial, and ethnic disparities in pain treatment that historically exist in our society [54, 55]. Similarly, in a separate study evaluating the impact of PDMPs in Kentucky, Ohio, and West Virginia, three states that had some of the earliest mandates for PDMPs utilization for providers, there were varying effects on prescribing patterns, though mandates did appear to increase PDMPs registration and utilization [56]. Furthermore, another study found that PDMPs implementation without additional mandatory provider access requirements had no significant impact on prescribing trends [21]. The lack of significant policy impact observed in our study is consistent with evidence that state‐level opioid prescribing restrictions have limited real‐world effectiveness [41].

In specific post‐operative settings, PDMPs implementation has demonstrated effectiveness in reducing opioid prescribing patterns [57, 58, 59]. In one study, evaluating opioid prescribing patterns after mandatory PDMPs consultation was implemented in a large multi‐site healthcare system, there were statistically significant reductions in both MMEs and median number of tablets prescribed following general surgery and orthopedic procedures; however, this decrease in MMEs was not clearly demonstrated in obstetric patients undergoing CD [57]. Similarly, another study looking at opioid prescription patterns in the pediatric population post‐surgery noted a decrease in the rate of opioid prescriptions per month and reduction in opioid prescriptions of more than 5 days following PDMP implementation [58]. Reassuringly, patient reported outcomes did not worsen after PDMPs use mandates after surgery [59]. However, these populations differ significantly from our study cohort and involved various types of surgeries, making their findings not entirely applicable to the postpartum population. Furthermore, these studies evaluated the effects of PDMPs after state and institutional level use mandate, which vary considerably across states in their scope and requirements (e.g., applying only to schedule II–IV opioids such as oxycodone, fentanyl etc.) [60], and not reflective of the impact of PDMPs implementation alone. Incorporating lessons from earlier programs, implementing PDMPs with stronger clinical features designed to improve real‐time prescribing decisions and broaden applicability beyond chronic care has the potential for greater reductions in prescribing patterns [61].

There are limitations with the current study that can be expanded upon in future research. First, our database primarily consists of commercially insured patients, overrepresenting privately insured individuals, as 40% of pregnant and postpartum patients have Medicaid coverage [2, 62, 63]. Therefore, our findings may not be generalizable beyond the commercially insured population. The comparison between commercially insured patients and Medicaid patients was not due to an imbalance in the populations included in our study. Second, our statistical balanced panel design was limited by the dataset period (2006–2015), which prevented longer periods of observation before and after PDMP implementation. This reduced our capacity to detect policy effects over an extended lag time. Third, race and ethnicity information was not available in our dataset, which limited our ability to assess whether PDMP implementation had differential effects across racial and ethnic groups [54, 55]. The inclusion of race and ethnicity data in future studies would allow for a more precise evaluation of potential disparities in PDMP policy effects. Fourth, there may be external influences impacting prescriber patterns that cannot be evaluated or accounted for in this population.

Further research should aim to replicate these findings in a multi‐payer nationally representative population and assess the long‐term impact of PDMPs on opioid prescribing. Additionally, subsequent studies should evaluate how variation in state PDMP mandates influences prescribing behaviors, as mandates differ in scope (e.g., applying only to schedule II vs. schedules II–IV medications) and may exert stronger effects than PDMP implementation alone [56, 64]. Studies should examine national policies, such as the 2016 CDC guideline [14], and their effects on postpartum opioid prescribing. These investigations are important in ultimately determining the effectiveness of such strategies in mitigating adverse patient outcomes such as the development of opioid misuse disorder and overdose in the pregnant and postpartum population.

In this study, state‐wide PDMPs implemented before 2016 did not have a significant impact on the immediate postpartum opioid prescribing patterns across states. These findings suggest that PDMPs alone may be insufficient to influence opioid use in the postpartum period.

## Funding

The authors have nothing to report.

## Conflicts of Interest

The authors declare no conflicts of interest.

## Supporting information


**Table S1:** ICD‐9 and ICD‐10 diagnosis and procedure codes are used to identify pregnancies.
**Table S2:** ICD‐9 and ICD‐10 diagnosis and procedure codes are used to identify deliveries.
**Table S3:** ICD‐9 and ICD‐10 codes are used to classify vaginal vs. cesarean deliveries.
**Table S4:** List of opioid analgesics included in the final analytic cohort.
**Figure S1:** Schematic of the study design.
**Figure S2:** Flowchart depicting inclusion and exclusion criteria for the final analytic cohort.
**Figure S3:** Schematic of the multiple pregnancy logic.
**Table S5:** ICD‐9 and ICD‐10 diagnosis codes used to identify OUD.
**Table S6:** Year of Prescription Drug Monitoring Program (PDMP) implementation by state.
**Methods:** Difference‐in‐Differences Specification.
**Table S7:** ICD‐9 and ICD‐10 Diagnosis Codes Used to Identify Medical Comorbidities.
**Table S8:** Full Covariate‐Adjusted Difference‐in‐Differences Estimates for Low‐Risk Pregnancy Episodes.
**Table S9:** Full Covariate‐Adjusted Difference‐in‐Differences Estimates for High‐Risk Pregnancy Episodes.
**Table S10:** Full Unadjusted Difference‐in‐Differences Estimates for Low‐Risk Pregnancy Episodes.
**Table S11:** Covariate‐Adjusted Difference‐in‐Differences Estimates for Low‐Risk Pregnancy Episodes (≥ 90 MME per Day Claims Model).
**Table S12:** Covariate‐Adjusted Subgroup Difference‐in‐Differences Estimates for Vaginal Deliveries in Low‐Risk Pregnancy Episodes.
**Table S13:** Covariate‐Adjusted Subgroup Difference‐in‐Differences Estimates for Cesarean Deliveries in Low‐Risk Pregnancy Episodes.
**Table S14:** Full Unadjusted Difference‐in‐Differences Estimates for High ‐Risk Pregnancy Episodes.
**Table S15:** Covariate‐Adjusted Difference‐in‐Differences Estimates for High ‐Risk Pregnancy Episodes (≥ 90 MME per Day Claims Model).
**Table S16:** Covariate‐Adjusted Subgroup Difference‐in‐Differences Estimates for Vaginal Deliveries in High‐Risk Pregnancy Episodes.
**Table S17:** Covariate‐Adjusted Subgroup Difference‐in‐Differences Estimates for Cesarean Deliveries in High‐Risk Pregnancy Episodes.

## Data Availability

The data that support the findings of this study are available from IQVIA. Restrictions apply to the availability of these data, which were used under license for this study. Data are available from the author(s) with the permission of IQVIA.
