# Supplementary material for: Impact of Prescription Drug Monitoring Programs on Postpartum Opioid Prescribing Practices
Source: Health Serv Res. 2026 May 4;61:e70123. doi: 10.1111/1475-6773.70123 (PMC13136944; doi:10.1111/1475-6773.70123)
Supplement: Supplementary file 1 — Table S1: ICD‐9 and ICD‐10 diagnosis and procedure codes are used to identify pregnancies. Table S2: ICD‐9 and ICD‐10 diagnosis and procedure codes are used to identify deliveries. Table S3: ICD‐9 and ICD‐10 codes are used to classify vaginal vs. cesarean deliveries. Table S4: List of opioid analgesics included in the final analytic cohort. Figure S1: Schematic of the study design. Figure S2: Flowchart depicting inclusion and exclusion criteria for the final analytic cohort. Figure S3: Schematic of the multiple pregnancy logic. Table S5: ICD‐9 and ICD‐10 diagnosis codes used to identify OUD. Table S6: Year of Prescription Drug Monitoring Program (PDMP) implementation by state. Methods: Difference‐in‐Differences Specification. Table S7: ICD‐9 and ICD‐10 Diagnosis Codes Used to Identify Medical Comorbidities. Table S8: Full Covariate‐Adjusted Difference‐in‐Differences Estimates for Low‐Risk Pregnancy Episodes. Table S9: Full Covariate‐Adjusted Difference‐in‐Differences Estimates for High‐Risk Pregnancy Episodes. Table S10: Full Unadjusted Difference‐in‐Differences Estimates for Low‐Risk Pregnancy Episodes. Table S11: Covariate‐Adjusted Difference‐in‐Differences Estimates for Low‐Risk Pregnancy Episodes (≥ 90 MME per Day Claims Model). Table S12: Covariate‐Adjusted Subgroup Difference‐in‐Differences Estimates for Vaginal Deliveries in Low‐Risk Pregnancy Episodes. Table S13: Covariate‐Adjusted Subgroup Difference‐in‐Differences Estimates for Cesarean Deliveries in Low‐Risk Pregnancy Episodes. Table S14: Full Unadjusted Difference‐in‐Differences Estimates for High ‐Risk Pregnancy Episodes. Table S15: Covariate‐Adjusted Difference‐in‐Differences Estimates for High ‐Risk Pregnancy Episodes (≥ 90 MME per Day Claims Model). Table S16: Covariate‐Adjusted Subgroup Difference‐in‐Differences Estimates for Vaginal Deliveries in High‐Risk Pregnancy Episodes. Table S17: Covariate‐Adjusted Subgroup Difference‐in‐Differences Estimates for Cesarean Deliveries in High‐Risk Pregnancy Ep [file HESR-61-0-s001.docx]

**Supplementary Analysis**

**Supplemental Table 1.** ICD-9 and ICD-10 diagnosis and procedure codes are used to identify pregnancies

**Supplemental Table 2.** ICD-9 and ICD-10 diagnosis and procedure codes are used to identify deliveries

**Supplemental Table 3.** ICD-9 and ICD-10 codes are used to classify vaginal vs. cesarean deliveries

**Supplemental Table 4.** List of opioid analgesics included in the final analytic cohort

**Supplemental Figure 1.** Schematic of the study design

**Supplemental Figure 2.** Flowchart depicting inclusion and exclusion criteria for the final analytic cohort

**Supplemental Figure 3.** Schematic of the multiple pregnancy logic
**Supplementary Table 5:** ICD-9 and ICD-10 diagnosis codes used to identify OUD

**Supplemental Table 6.** Year of Prescription Drug Monitoring Program (PDMP) implementation by state

**Supplementary Methods:** Difference-in-Differences Specification

**Supplementary Table 7:** ICD-9 and ICD-10 Diagnosis Codes Used to Identify Medical Comorbidities

**Supplementary Table 8:** Full Covariate-Adjusted Difference-in-Differences Estimates for Low-Risk Pregnancy Episodes

**Supplementary Table 9:** Full Covariate-Adjusted Difference-in-Differences Estimates for High-Risk Pregnancy Episodes

**Supplementary Table 10:** Full Unadjusted Difference-in-Differences Estimates for Low-Risk Pregnancy Episodes

**Supplementary Table 11:** Covariate-Adjusted Difference-in-Differences Estimates for Low-Risk Pregnancy Episodes (≥90 MME per Day Claims Model)

**Supplementary Table 12:** Covariate-Adjusted Subgroup Difference-in-Differences Estimates for Vaginal Deliveries in Low-Risk Pregnancy Episodes.

**Supplementary Table 13:** Covariate-Adjusted Subgroup Difference-in-Differences Estimates for Cesarean Deliveries in Low-Risk Pregnancy Episodes.

**Supplementary Table 14:** Full Unadjusted Difference-in-Differences Estimates for High -Risk Pregnancy Episodes

**Supplementary Table 15:** Covariate-Adjusted Difference-in-Differences Estimates for High -Risk Pregnancy Episodes (≥90 MME per Day Claims Model)

**Supplementary Table 16:** Covariate-Adjusted Subgroup Difference-in-Differences Estimates for Vaginal Deliveries in High -Risk Pregnancy Episodes.

**Supplementary Table 17:** Covariate-Adjusted Subgroup Difference-in-Differences Estimates for Cesarean Deliveries in High-Risk Pregnancy Episodes.

**Supplemental References**

**Supplemental Table 1. ICD-9 and ICD-10 diagnosis and procedure codes are used to identify pregnancies**

Pregnancies were identified in the IQVIA claims database using International Classification of Diseases, Ninth Revision (ICD-9) and Tenth Revision (ICD-10) diagnosis and procedure codes. Code selection was guided by previously published papers,^1^ and refined through input from a clinical expert and practicing obstetrician-gynecologist.

| ICD - 9 | ICD-10 |
| --- | --- |
| 651.30, 645, 646.5, V28.9, 646.20, V28.2, 765.24, V72.42, 640.83, 765.22, 760.64, 651.10, 646.13, 643.8, 765.21, 640.80, V22.0, 633.1, 651.41, 651.1, V23.81, 642.33, 760.63, 651.20, 651.03, 646.93, 643.83, 651.40, V23.4, 633.2, V72.41, 646.73, 651.00, 646.1, 659.63, 646.83, 649.51, 649.50, 649.53, 761.8, 761.5, 646.91, 646, 633.10, 645.11, 761.9, 651.5, 765.20, 651.23, 643.93, 640.90, 646.50, 659.5, 646.63, 633.00, V23.49, V28.8, 646.21, 645.2, 643.9, 651.53, 646.90, 643.21, V23.7, V28.89, V28, V22.1, 645.1, 659.4, 645.13, V28.4, 633.20, 646.81, 640.9, V15.21, 646.7, 659.60, 642.3, 643, 645.10, 649.5, 765.26, 659.50, 760, 646.80, 643.80, 761, 646.10, 646.2, 631.0, V61.6, V23.5, 651.11, 646.9, V23.9, 646.40, 765.25, 633.01, 659.51, 651.43, V23.86, V61.7, 633.21, 643.20, 640.8, V23.87, 765.28, V23.1, V23.84, 645.21, 646.61, V22, 765.23, 646.70, 765.29, 651.61, 646.43, 646.23, 640.81, 765.2, 646.11, 646.4, 646.8, 640, V23, 646.60, 659.53, V28.0, V28.6, 646.6, 645.20, 651.60, V28.5, 646.71, V23.0, 646.41, 643.81, 651.21, V25.04, 671.9, 643.2, V23.83, 640.93, V23.89, 646.51, 651.4, V23.3, V23.8, 651.6, 651.13, V23.85, 633.0, 651.3, 659.61, 651.31, 643.91, 765.27, 651.01, 659.43, 633.11, 642.30, 642.31, 651.63, 646.53, 651.33, 643.90, 651.2, V23.41, V23.82, 651.50, 640.91, 643.23, 651.51, V28.1, 796.5, 645.23, 651.0 | O41.03X0, O10.913, O36.8120, O30.011, O43.013, O41.92X1, O41.03X3, O36.192, O36.62, O36.5930, O09.211, O99.333, O26.92, O44.01, O36.90X0, O30.092, O36.23X0, O98.619, O30.10, Z3A.29, O44.12, O36.1930, O31.30X1, O41.1223, O46.013, O30.1, O30.109, O22.33, O98.712, O9A.212, O36.8921, O40.1XX0, O36.8330, O31.33X1, O46.019, O24.410, O99.019, O36.011, O29.299, O31.8X24, O88.21, O09.219, O11.3, O36.8132, O25.11, O88.819, O98.111, O9A.111, O00.21, O00.212, O31.03X0, O29, O42.013, Z3A.01, O00.0, O26.52, O91.211, O23.522, O36.90X3, O29.022, O98.91, O43.813, O16.3, O45.90, O43.192, O36.0112, O43.023, O34.62, O42.911, O29.012, O36.8394, O98.513, O31.8X32, O9A.211, O31.8X11, O41.8X31, O36.73X2, O99.71, O44.03, O40.1XX3, O41.1213, O88.019, O36.23X2, O88.81, O29.3X3, O36.8920, O98.911, O22.21, O98.811, O36.8232, O41.1032, O44.30, O9A.513, O09.892, O23.40, O09.31, O36.891, O43.91, O36.0923, O41.122, O36.8392, O36.1111, O36.20X2, O31.00X9, O99.213, O99.323, O41.8X20, O40.3XX3, O36.8123, O36.111, O46.92, O28.0, O36.5122, O41.8X10, O31.20X1, O42.011, O88.01, O36.513, Z34.91, O36.0120, O26.50, O41.1021, O36.1113, O46.93, O31.8X39, O22.00, O40.1XX2, O46.002, O36.8313, Z3A.2, O36.0920, O43.819, O36.0191, O11.1, O26.821, O41.1492, O36.1912, O41.149, O41.90X3, O45.091, O36.80, O31.23X5, O36.0131, O98.211, O22.8X1, O99.413, O9A.311, O44.20, O36.8212, Z34.0, O36.829, O30.113, O44.13, O24.919, O34.70, Z3A.26, O36.8910, O36.91X0, O09.621, O31.8X30, O31.23X0, O26.649, O41.143, O36.91, O23.9, O26.91, Z3A.49, O22.41, O34.599, O99.711, O36.8932, O9A.31, O09.521, O99.210, O92.013, O36.5933, O71.03, O36.8922, O41.8X21, O13.4, O36.1191, O40.1, O30.042, O00.1, O34.83, O12.13, O47.03, O36.593, O23.11, O30.119, O71.00, O36.1112, O29.092, O10.013, O26.842, O88.119, O46.003, O98.41, O36.191, O09.3, O29.9, O31.8X15, O43.193, O09.213, O36.5910, O31.8X21, O36.091, O36.23X1, O10.119, O88.013, O98.019, O29.023, O29.29, O34.02, O34.81, O46.8X3, O36.5133, O10.211, Z36.4, O36.71, P01, O99.113, O26.832, O36.819, O10.412, O36.20X0, O45.91, O98.212, O36.0932, O36.8393, O31.8X12, O22.10, O31.8X90, O10.312, O41.1033, O23, O30.039, O36.93X1, O36.72X0, O36.93X3, O16.9, Z3A.00, O10.919, O36.5913, O09.70, O00.2, O22.51, O45.009, O41.1433, O36.8292, O23.20, O36.8233, O36.72X3, O41.1230, O36.8131, O30.049, P01.5, O36.5923, O88.811, O91.111, O41.129, O45.012, O30.199, O9A.411, O41.101, O36.821, O22.5, O24.311, Z3A.31, O28.5, O26.31, O36.8193, O29.211, O92.119, O09.42, O34.519, O36.0113, O99.01, O46.012, O36.8315, O23.43, O45.93, O98.912, O26.13, O41.00X1, O31.33X0, O15.00, Z36.84, O36.8192, O36.90X2, O36.20, O24.011, O36.1110, O36.0132, O41.8X92, O98.11, O23.3, O45.011, O91.21, O26.873, O26.892, O26.853, O36.5121, O98.413, O98.119, O09.A3, O29.219, O41.00, O29.1, O09.33, O26.841, Z3A.18, O09.812, O36.63X3, O14.00, O26.32, O36.0123, O41.1293, O31.22X4, O36.92, O26.893, O42.113, O36.71X0, O98.611, O43.232, O31.32X4, O31.32X5, Z3A.11, Z3A.27, O09.293, O09.511, O99.112, O09.91, O36.0121, Z64.0, O43.121, O31.22X1, O98.51, O98.71, O26.9, O12, O99.411, O36.5990, O34.521, O36.8220, O36.72X1, O36.822, O41.1011, O29.119, O9A.112, O34.00, O36.019, O88.011, O29.5X2, O44.02, O36.512, O34.12, O43.112, O22.8X3, O30.111, O42.111, O9A.313, O31.02X3, O30.02, Z3A.19, O36.5931, O36.113, O30.019, O36.5192, Z3A.1, Z3A.3, O43.221, O36.013, O44.52, O41.01X3, O36.21X2, O88.319, O36.5111, O36.199, O29.111, Z34.9, O30.03, O36.72, O23.00, O45.8X2, O31.30X0, O43.239, O36.8391, O22.1, O36.1992, O31.22X5, P00, O98.311, O36.8191, O31.22X2, O45.029, O00.209, O98.012, O30.133, O31.8X93, O43.233, O36.823, O29.3X1, Z34.90, O09.90, O29.40, O36.73X0, O44.51, O00.112, O22.4, O41.141, O36.1920, O99.412, O22.03, O23.22, Z36.8A, O09.62, O26.81, O31.8X13, O29.8X9, O26.71, O99.519, O36.193, O22.13, O99.840, O98.411, O91.019, O22.9, O31.02X2, O29.5X3, O45.099, O31.00X5, O26.8, Z34.92, O34.92, O23.31, O88.312, O34.532, O9A.319, O88.112, O36.8334, O99.283, O41.8X3, O31.02X0, O24.81, O31.00X3, O31.31X5, Z3A.16, O26.611, O42.119, O36.1931, O43.229, O00.10, O36.22X2, Z36.89, O31.8X92, O09.893, O10.011, O40.3XX1, O34.40, O98.319, O44.22, Z36.2, Z36.82, O36.71X3, O31.00X1, O99.322, O28.4, O30.123, O12.11, O29.293, O92.019, O30.19, O41.02X0, O41.93X1, O34.91, O43.211, Z36.87, O31.03X1, O09.00, O26.899, O24.112, O36.1991, O36.8923, O36.8311, Z3A.38, O34.13, O23.592, O98.819, O26.839, O36.5120, O29.41, O23.523, O36.5112, O29.3X2, O47.0, O36.22X0, O24.812, O23.30, O00.201, O29.192, O29.5X9, O41.1413, O99.013, Z3A.34, O09.212, O30.102, O41.02X2, O36.012, O10.411, O36.0933, O9A.512, O41.1092, O36.80X0, O99.611, O98.312, O26.42, O36.8293, O99.41, O92.112, O36.91X3, O99.330, O26.813, O34.31, O09.41, O31.8X31, O98.613, Z36.5, O36.60X2, O31.8X29, O31.23, O26.872, O24.113, O99.280, O28.9, O36.519, O41.1210, O41.8X91, O23.529, O36.92X2, O09.519, O11.9, O36.8121, O43.109, O43.029, O24.11, O14.03, O44.00, O36.63X2, O36.91X2, O45.019, O26.84, O36.23X3, O09.523, Z34.80, O99.331, O30.099, O10.212, O29.90, O10.311, O09.A0, O36.8322, O36.1121, O36.71X2, O28.3, O10.912, O98.519, O36.8331, O29.61, O31.00X2, O36.63X0, O34.593, O10.911, O09.43, O40.2XX1, O36.8395, O31.31X9, Z3A.32, O09.292, O22.42, O98.412, O99.619, O22.91, O36.8390, O44.50, O29.8X3, O36.63, O41.1221, O36.8992, Z34.8, O36.73, P07.30, O43.123, O14.90, O12.23, O26.822, O34.512, O00.211, O29.213, O98.61, P07.20, O36.5921, O12.03, O31.32X1, O88.219, O88.311, O88.211, O36.8222, O40.2XX3, O9A.519, O10.21, O26.82, O26.891, Z3A.41, O99.343, O23.90, O43.899, O26.831, O30.023, O36.8912, O26.89, O00.219, O31.20X3, O41.1292, O09.9, Z3A.37, O99.340, O36.8133, Z3A.0, O44.43, O00.20, O31.31X2, O31.02X1, Z3A.12, O30.003, O23.01, O12.01, O46.093, O34.531, O29.91, O30.012, O34.41, O36.0922, O36.0911, O29.193, O30.009, O09.30, O46.8X9, O10.413, O30.191, O31.21X1, O29.011, O47.00, O34.43, O09.629, O30.112, O26.712, O34.511, O44.21, O36.833, O29.099, O36.119, O9A.213, O36.1910, P01.8, O30.022, O31.8X19, O36.8325, O34.80, O36.92X0, O23.23, O22.53, O09.2, O99.351, O99.842, O46.091, O41.91X3, O99.512, O41.1220, O40.1XX1, O41.8X11, O31.21X9, O31.01X1, O36.8319, O88.012, O36.8211, O9A.21, Z3A.10, O36.0910, O98.812, O46.8X2, O22.8X2, O36.22X3, O13.9, O26.859, O36.80X2, O48.1, O36.0993, O36.73X1, O88.313, O36.60, O23.2, O31.8X95, O30.031, O36.70X0, O10.313, O09.73, O23.59, O14.23, O36.5110, O31.33X2, O9A.119, O36.8329, O36.1133, O99.352, O09.71, O41.8X13, Z3A.09, O34.539, O91.013, O31.22, O46.001, O36.0991, O31.31X3, O10.41, O31.33, O98.919, O36.5912, O43.191, O31.33X3, O10.11, O99.11, O41.142, O26.30, O25.12, O36.8231, O36.8321, O99.342, O36.0190, O22.40, O36.592, O41.8X9, O31.23X9, O45.002, O31.03X2, O41.1212, Z34.83, O16.1, O36.5991, O41.8X2, O36.0192, O28.8, O40.9XX0, O30.041, O10.012, O43.93, O23.593, O24.319, O45.003, Z36.8, O20.9, O43.102, Z98.870, O24.813, O31.8X20, O22.90, O36.8993, Z34, O46.022, Z3A.13, O42.90, O28.2, O88.113, O09.51, O29.129, O13.3, O09.299, O43.012, O29.013, O41.121, O09.03, Z3A.33, O09.611, O42.913, O14.93, O09.92, O36.5131, O36.813, O26.833, O24.911, O99.712, O34.533, O41.91X1, O9A.312, O41.8X32, O43.231, O40.2XX2, O45.093, O98.713, O34.90, O98.213, O43.021, O41.8X90, O99.820, O30.103, O36.0122, O30.139, O46.099, O36.8312, O30.01, Z36, O36.8130, Z3A.23, O16.2, Z36.3, O36.62X3, O31.30X2, O09.82, O36.8930, Z3A.20, O43.892, O45.8X9, O36.61X2, O09.61, O98.313, O99.312, O9A.413, O41.1291, O09.813, O24.41, O99.212, Z3A.42, O31.21X3, O41.1432, O24.119, O34.32, O09.02, O43.122, O42.019, O31.31X4, O36.1122, O22.50, O92.011, O31.03X9, O29.029, Z34.81, O36.0930, O99.613, O24.415, O31.8X3, O30.132, O29.93, O99.843, O30.192, O99.310, O41.93X2, Z36.0, O23.03, O10.319, Z3A.4, O29.113, O30.0, O9A.51, O36.5920, O09.613, O10.019, O99.513, O43.223, O88.212, O22.11, O92.11, O29.8, O26.12, O44.11, O43.212, O36.60X1, O41.8X22, Z36.1, O31.8X10, O41.8X12, O34.513, O41.90X2, O43.90, O31.01X3, O34.71, O91.012, O36.1193, O41.1093, O26.851, Z3A.40, Z3A.15, O36.5911, O46.009, O44.42, O41.01X2, O41.00X2, O26, O09.93, O29.63, O41.102, Z34.01, O26.86, O41.03X1, O36.61X3, O99.713, O9A.219, O34.529, O09.72, O46.023, O99.61, Z3A.30, O24.913, O23.42, O36.0931, O40.9XX2, Z3A.28, O34.33, O43.022, O91.119, O10.111, O36.5993, O36.0913, O36.60X3, O36.8913, O00.119, O31.23X3, O31.8X94, Z3A.14, O26.812, O31.31X1, O29.8X1, Z3A.35, O21.8, O22.02, O36.21X1, O23.513, O36.8122, O41.1233, O24.312, O24.811, O9A.113, O31.21X2, O00.102, O41.1410, O21, O36.5130, O30.093, O41.90X0, O41.1232, O98.81, O36.1192, O43.113, O36.092, O99.311, O31.32X0, O23.21, O41.1421, O26.61, O31.33X4, O41.91X2, O26.643, O36.8223, O26.53, O40.2XX0, O41.1020, O26.41, O31.01X0, O31.20X2, Z3A.25, O09.291, O23.599, O09.821, O46.8X1, O34.93, O36.1131, Z3A.24, O36.8290, O36.8320, O36.70X1, O23.51, O41.1411, O47.02, O11.2, O36.892, O34.73, O26.711, O09.21, O98.31, O24.31, O31.21, O31.8X14, O34.42, O99.011, O36.1990, O98.21, O41.1091, O24.414, O36.5922, O22.32, O26.819, O44.53, O36.8332, O41.1010, O99.51, O23.10, O99.332, O24.912, O36.899, O36.5190, O34.63, O34.60, O29.199, O10.419, O41.01, O24.313, O98.511, O14.22, O88.213, O24.419, O26.93, O36.8911, O30.021, O36.0992, O91.11, O30.043, Z30.02, O12.10, O36.61X1, O31.8X23, O36.0193, O44.40, O26.40, O21.9, O99.111, O36.0921, O41.103, O36.8221, O36.8333, O29.091, O36.8190, O36.62X0, O23.32, O36.812, O36.62X1, O99.320, O99.321, O98.612, O31.21X4, O44.32, O15.02, O41.02, O26.1, O23.93, O20.8, O36.92X1, O12.12, O40.9XX1, O36.62X2, O43.101, O36.21X0, O36.1932, O10.112, O36.839, O36.8399, O41.109, O29.112, O92.01, O44.10, O30.013, O22.31, O31.22X3, O41.1422, O36.22, O91.011, O41.8X30, O43.92, O14.20, O36.511, O43.111, P01.9, O10.01, O48, Z3A.22, O26.02, O41.92X2, O36.71X1, O99.419, O12.20, O41.1031, O44.31, Z36.86, O31.23X4, O41.1013, O26.811, O02.81, O36.61X0, O36.8931, O45.8X3, O41.123, O25.13, O30.11, O15.03, O26.613, O28.1, O46.91, O13.2, O31.21X0, O71.02, O36.0912, O00.101, O45.022, O31.33X9, O31.01X2, O30.029, O31.20, O22.0, O09.6, O36.831, O36.93, O44.33, O91.113, O22.12, O29.92, O43.891, O45.92, O41.1430, O43.199, O26.85, O30.04, O24.819, O36.5132, O36.1922, O23.1, O31.30X3, O29.093, O41.8X23, O41.91X0, O26.829, O26.10, O41.03X2, O98.719, O34.523, O00.109, O91.212, O26.00, O91.219, O00.00, Z36.88, O31.30X9, O34.01, O99.012, O14.02, O26.51, O43.219, O26.01, O31.8X2, O41.93, O26.843, O26.11, O42.012, O30.122, Z34.02, O30.032, O99.810, O99.830, O23.33, O36.8991, O36.72X2, O43.222, O41.1222, O9A.412, O22.8X, O99.119, Z3A.08, O23.591, O36.0130, O26.852, O14.10, O12.02, O45.013, O31.8X91, O22, Z34.93, O36.893, O36.0133, O09.623, O31.31X0, O36.8339, O09.512, O42.112, O99.282, O42.00, O34.10, O98.913, O98.112, O34.61, O88.111, O36.112, O36.73X3, O23.0, O41.1023, O36.23, O46.021, O13, O26.642, O41.90, O23.02, O99.353, O42.919, O98.113, O00.11, O98.013, O36.20X3, O31.8X9, Z3A.39, O09.619, O41.01X1, O41.8X1, O41.8X33, O31.8X1, O36.8324, O98.01, O98.512, O41.01X0, O99.719, O30.121, O31.32X3, O31.8X99, O31.03X3, O09.522, O23.91, O34.30, O36.1190, O45.8X1, O43.019, O22.92, O29.291, O36.8314, O30.09, O91.01, O41.1030, O26.823, O92.113, O46.029, O29.3X9, O36.80X1, O44.41, O00.111, O36.90X1, O36.5191, O31.01X9, O36.92X3, O00.01, O91.112, O24.013, O14.92, O10.91, O23.521, O09.A1, O9A.419, O41.1012, O31.30X4, O26.619, O09.513, O36.5932, O10.219, O36.1921, O36.5992, Z36.81, O29.62, O21.2, O09.52, O36.5113, O41.93X3, O14.12, Z3A.21, O36.22X1, O36.1130, O26.33, O24.01, O31.20X9, O23.52, O26.90, O25.10, O31.00X0, O31.22X9, O92.012, Z34.82, O88.812, O26.719, O24.019, O99.350, O43.129, O44.23, O23.519, O36.5123, O41.00X3, O99.891, O31.8X34, O40.9XX3, O36.1923, O47.1, O24.012, O99.341, O22.43, O09.822, O31.23X1, O36.8291, O36.70X3, O23.13, O10.113, O98.419, O36.80X3, Z36.9, O09.829, O36.1993, O41.92X3, O43.103, O30.033, O36.0990, O42.912, O41.1090, O42.10, O22.01, O23.92, O99.841, O26.43, O36.91X1, O36.20X1, O92.111, O23.5, Z3A.17, O30.001, O34.591, O41.1231, O31.23X2, O91.213, O30.00, O22.23, O09.811, O36.591, O22.20, O41.90X1, O12.21, O41.8X93, O36.21, O40.3XX0, O26.849, O41.1022, O29.8X2, O29.292, O29.212, Z3A, Z34.00, O23.41, O09.823, O98.011, Z36.85, O36.63X1, O41.93X0, O22.22, O34.522, O36.832, O36.1132, O36.90, O41.02X1, O34.03, O29.5X1, O41.1431, O41.1420, O31.21X5, O29.191, O09.899, O41.02X3, O34.11, O36.093, O36.1911, O23.512, O30.193, O45.023, O09, O30.101, O12.00, O99.612, O41.1290, O43.119, O29.60, O36.70X2, O41.00X0, O22.30, O36.5193, O36.1123, O36.8990, O26.713, O43.811, O12.22, O46.011, O36.93X0, O36.0111, O30.131, O20, O36.1913, O46.90, O09.612, O36.599, O43.011, O31.8X33, O41.1211, O41.1490, O26.879, O13.1, O36.1120, O31.8X22, O23.4, O29.123, O45.021, O98.813, O23.12, O30.091, O29.43, O26.83, O30.12, O24.111, O36.60X0, O09.40, O98.711, O36.93X2, O22.52, O24.91, O26.641, O99.281, O40.3XX2, O26.612, O09.622, O31.22X0, O31.32X9, O9A.511, O22.8X9, O36.61, O36.8335, O14.13, O09.819, O09.29, O00.202, O36.8323, O48.0, O43.893, O09.32, O41.92X0, O9A.11, O22.2, O09.891, O88.813, O36.8230, O46.092, O36.8210, Z3A.36, Z34.03, O99.313, O09.A2, O31.32, O09.529, O99.511, O09.01, O99.211, O31.20X0, O36.0110, O10.213, O29.42, O41.03, O29.121, O26.03, O30.002, O98.219, O36.099, O36.8310, O36.8213, O29.019, O88.11, O34.592, O43.812, O23.511, O31.32X2, O34.72, O36.1933, O36.8933, O30.129, O25.1, O09.81, O43.213, O34.82, O22.93, O45.092, O45.001, O09.5, O40.3, O41.1491 |

**Supplemental Table 2. ICD-9 and ICD-10 diagnosis and procedure codes are used to identify deliveries**

Deliveries were identified in the IQVIA claims database using International Classification of Diseases, Ninth Revision (ICD-9) and Tenth Revision (ICD-10) diagnosis and procedure codes. Code selection was guided by previously published papers,^1^ and refined through input from a clinical expert and practicing obstetrician-gynecologist.

| ICD - 9 | ICD-10 |
| --- | --- |
| V31.01, V27.9, V39.2, 777.3, 760.3, 660.9, 658.23, 760.1, 662.20, 669.14, V32.2, 770, V27.0, 669.12, 663.9, 649.8, 669.11, 762.0, V37.01, 663.50, 669.20, 663.53, 779.33, 669.02, 669.50, 760.64, 663.6, V37.2, 769, 653.1, 660.2, 775, 662.3, 663.63, V39.1, 763.82, V27.7, 668.92, V29, V34.0, 669.70, 762.7, 668.84, 772.0, 660.00, 772.1, V39, 665.11, 659.21, 779.0, 763.1, 642.42, V30.0, 659.83, 760.74, 760.61, 778.5, 658.20, 771.81, 663.80, V27.3, 663.91, 777.51, V31.0, 762.9, 644.03, 760.63, 660, 668.0, 770.5, 777.52, 659.30, 664.5, 772.8, 662.1, 668.21, 778, V33.01, 669.83, 668.9, 660.8, 662.33, 663.81, V31.00, 775.9, V35.01, 776.0, 668.8, 770.3, 768.2, 648.62, V24.0, 659.8, 663.00, 773.4, 664.64, 779.34, 669.32, 661.90, 660.93, 763.81, 777.53, 761.8, V36.00, 668.14, V36.2, 653.9, V20.31, 761.5, 763.8, 772.9, 668.11, V30.01, 669.82, 669.00, 664.3, V34, V32.0, 770.84, 762.1, 664.1, V34.00, 669.84, 762, V37.0, 668.91, 774.2, 661.31, 761.9, 776.2, 653.8, 662.21, 659.90, 775.7, 659.80, 774.7, 662.10, V20.32, V30.2, V39.00, 663.13, V27, 658.31, 663.93, 668.82, 659.33, 760.5, 762.4, 778.3, 664.8, 773.5, 658.2, 669.13, 760.79, 772.4, V37, 663.51, 669.91, 665.1, 660.10, V37.1, 662.31, 760.78, 760.72, 663.23, 658.30, 644.0, 663.03, 668.1, V27.1, 659.23, 668.24, 650, 660.01, 779.3, 762.8, 665.03, 644, 661.3, 763.4, 662.11, 669.90, 770.87, 642.62, 668.02, 779.2, 761.1, 663.1, 644.21, 761.2, 663.5, 653.2, 644.13, 770.82, 649.82, 642.72, V30, 779.31, 778.2, 663.8, 669.94, V35.1, 648.22, V37.00, 770.9, 777.4, 761.0, 669, 761.4, 669.93, 773.2, 661.91, 668.80, 659.9, 779.4, 662.00, V27.4, 663.40, 777.5, 653.0, 762.6, 773.0, 760.77, 668.90, 776.9, 659.93, 762.3, 660.91, 659.3, 770.83, 763.6, 760, 760.2, 668.81, 660.20, 644.2, 647.92, V27.6, 761, 668.22, 659.2, 658.21, 648.42, 779.85, 662, 642.52, 778.8, V32.1, V28.82, 669.7, 668.00, 649.81, 669.04, 763.2, 660.81, V33.2, V35.0, V33.00, 664.9, V39.01, V31.1, 658.3, 661.33, V36, 768.3, 644.10, 778.7, 642.32, 777.2, 771.83, 663.83, 775.81, 659.31, 664.2, V36.0, V34.1, V30.1, 648.12, V31.2, 644.1, 779.32, 660.3, 663.4, 779.5, 760.76, 668.03, 669.51, 778.4, 648.82, 668.13, 762.2, 668.23, 660.4, 782.4, 659.81, 663.90, 664.60, 648.92, 664.6, 770.2, V32.01, 669.80, 669.10, 668, 661, 760.71, V34.2, 665.0, 663.11, 760.0, 772.6, 763.3, 646.82, 660.13, 662.30, 770.1, 668.04, 648.52, 669.81, 669.71, 760.4, 665.00, 668.20, 644.20, V36.01, 669.5, 770.18, 663.31, V27.5, 763.9, 660.0, 668.2, 647.52, 763.84, 760.8, 663.20, 668.12, 770.17, 660.11, 669.03, 772.2, V33.0, 661.9, 663.41, 663.2, 647.82, V33, V34.01, 663.33, 778.9, 663.61, 669.8, 776, 660.23, V36.1, 660.83, 763.89, 760.9, 669.92, 763.0, 768.9, 665.4, 763.5, 660.80, 663, 775.4, V29.2, 770.6, V20.3, 660.1, 664, 668.93, 770.10, 662.2, 669.01, V39.0, 648.32, V30.00, 768.4, 662.23, 772.5, 669.34, 668.94, 771.82, 770.4, 663.3, 760.70, 668.10, V35.2, 760.75, 760.73, 664.61, 773, 659.91, V35, 770.81, 660.90, 763, 644.00, V27.2, 663.43, 664.4, V13.21, 662.13, 659.20, 663.60, 647.62, 662.0, 777.1, 658.33, 669.9, V32, 663.01, 662.01, 648.72, 659, 663.21, 661.30, 763.7, 665.01, 660.03, 773.1, V31, 762.5, V33.1, 647.42, 668.83, 662.03, 663.10, 653.3, 777.50, 669.3, V32.00, V35.00, 669.30, 771.4, 776.8, 663.30, 661.93, 668.01, 761.7, 760.7, 664.0, 646.52, 761.3, 663.0, 760.62, 660.21, 646.42, 770.88, 779.1, 646.62 | P02.7, P80.8, P92.01, O69.4XX1, O66.6, O15.1, P74.6, Z05.2, O75, O70.21, O60.10X3, P52.0, O75.82, P02.6, O42.013, P76.8, O60.10X2, P03, P05.07, P81.0, O42.911, O86.2, P92.8, O75.1, P04.11, O70, O69.1XX0, O64.0XX1, Z00.1, O70.2, P51.9, O64.9XX0, P19, P04.0, O86.22, P07.3, O42.0, O42.011, O74.3, P03.5, O69.5XX2, P50.2, O64.8XX1, P05.0, P05.10, P52.8, O70.20, P28.42, O69.9XX1, P28.0, O65.4, P83, P81.8, O64.4XX2, O60.14X2, O69.2XX1, O71.03, O42.01, P05.05, P70, P50.0, O71.00, O69.9, O42.02, P58.42, O69.1XX3, Z05.1, O64.5XX2, P00.81, O66, P01, P28.9, O62.8, P07.15, P74.4, O69.4XX2, P01.7, O60.12X3, O42.11, P01.5, P36.2, O69.0, O60.10X1, O65.3, O77, P07.34, P05.04, P92.1, P00.1, P92, P80.9, O60.14X0, P28.19, O64.3XX1, O70.23, O69.82X3, O75.81, P36.39, O75.9, O64.4XX0, O42.113, O69.8, P02.0, Z38.62, O65.8, O69.0XX1, O77.0, O63.9, P02.1, O63.0, P07.01, P00.4, Z05, O67, O42.111, Z05.41, P74.9, O70.4, P02.69, P94.8, P52.22, P05.08, O70.0, O69.5XX3, P00, O60.13X2, O60.12X1, P07.22, P70.9, Z38.2, O70.3, P04.9, O74.8, P04.1A, P74.0, O65.9, P05.13, O60.13X3, O65.2, Z37.2, P05.03, O69.9XX2, P00.2, P05.17, O42.119, O64.9XX3, P07.35, P36.0, Z05.42, O74.1, O60.14X3, Z05.72, P50.1, P28.39, P07.16, O75.8, P07.2, P50.9, Z05.43, P29.4, Z87.61, Z00.110, O64.3XX0, P91.9, P96.2, P05.09, P08.1, O60.12X0, O86.29, O86.12, P74.3, O65.1, P07.38, Z38.61, P05.2, P50.3, O60.22, P04.1, P07.33, O69.82X1, P28.5, O63.2, Z37, P28.1, O60.10X0, P77.3, O60.20X2, O90.41, P28.2, O69.2XX2, P74.31, P28.33, P07.26, Z37.0, P07.30, P04.19, P07.20, P05.06, P00.5, P04.6, P28.49, P92.2, O60.13X0, O69.89X2, P53, O69.1, P00.89, O64.9XX1, Z00.111, P19.1, P50.4, P28.8, P04.2, Z38.30, P52.3, P19.0, P96.3, P28.32, O74.5, P19.9, P01.8, P02.20, P05.19, P78.82, P07, P07.10, P92.3, P22, P07.17, O69.2, P03.81, O66.0, O69.81X2, Z05.6, O60.22X2, P05.15, P92.09, O63.1, P76.9, P02.29, P02.78, P04, O70.1, O64.9XX2, Z38.0, P74.22, P36, O75.2, L08.82, Z05.3, O69.81X3, O86.19, N47.0, P52.5, O60.13X1, O74.6, O64.2XX0, P38, O69.81X1, P03.3, O65.0, P51.0, P50.5, P02.2, O75.89, P55.0, P01.2, P04.17, P83.6, O64.1XX0, P03.82, Z00.11, P83.30, Z37.1, Z05.0, P52.21, P36.5, P29.30, O42.90, P36.30, O74.9, O76, P55.1, P55.8, O42.913, P08.21, P03.6, P02.4, O64.0XX3, O60.13, Z38.01, P22.1, O69, P02.8, P07.1, P07.21, Z05.73, P05.18, O64.0XX0, P05.11, P92.0, P05.1, O69.4, O69.3XX3, O64.1XX2, P04.49, O42.92, O86.21, P91.1, O42.019, P04.81, P28.89, P00.6, P91.88, P02.70, P07.18, P81.9, P05.02, P04.5, O90.0, P92.4, O66.8, P04.14, P22.8, O69.82X2, P55.9, O66.9, O68, P04.3, P76.1, O69.0XX2, P52.2, P03.810, O69.0XX3, P51, P77.1, P04.15, P74.32, P74.422, O74.4, P07.25, P07.0, P04.40, O64.4, P36.8, O60.12X2, P83.8, P03.1, Z05.8, Z62.813, O60.2, O60.22X3, P74.2, P83.88, Z38.3, P07.24, O75.3, O86.11, Z05.71, P36.9, Z87.51, P02, P05.00, P02.3, P29.81, O62, P93.8, P04.13, P28.31, O69.89X0, O70.9, P77.2, O60.22X1, P74.5, O64.1XX3, O60.23, O69.89X1, P05.16, O80, P05.01, O69.3XX1, O64.0XX2, P07.02, O74.2, P05.14, O66.2, O69.89X3, P28.81, P04.89, O64, P28.30, P52.9, P00.82, O63, P07.31, P28.43, P05, P01.9, Z05.9, P07.14, O60.23X2, P00.9, O77.1, P74.1, O69.0XX0, O71.02, O69.81, P81, O77.8, Z37.3, P03.89, P95, Z38.4, Z37.9, O74.0, P05.9, P90, P07.23, O69.2XX0, O60.14X1, P03.9, O64.8XX3, O69.82X0, P28.40, O42.12, O69.5XX1, O60.14, O64.3XX2, P28.4, O42.012, P83.4, P83.9, P52.6, P28.41, P03.2, O71.1, O64.4XX1, O60.10, P59.0, P02.5, O86.20, Z38.31, P28.3, O75.0, Z38.5, P39.2, O69.1XX2, O69.9XX3, Z05.81, O75.5, P28.10, P70.8, P01.4, O69.3XX2, P04.16, P22.0, P07.03, O42.112, O42.00, P00.3, O69.82, P92.9, O69.2XX3, O42.919, P74.49, Z38.1, P50, P92.6, P03.4, O64.1XX1, P55, O86.4, O70.22, O64.0, P04.42, P52.4, O64.2XX2, O60.20X3, P07.32, O66.3, P83.39, O77.9, P04.12, P05.12, O60.22X0, O60.20X1, O69.3, Z05.5, O69.89, P03.811, Z38.00, P36.4, O64.8XX2, O69.9XX0, O60.20, P01.3, O69.4XX0, O62.3, P51.8, P78.83, P94.9, P04.18, P52.1, O42.912, O42.10, P03.819, O42.91, P08.22, O69.3XX0, O60.23X3, O69.81X0, O82, P74.8, O60.23X1, P71.0, P50.8, P07.39, P00.0, P36.10, P01.1, O64.2, P07.36, P96.5, P08.0, P04.8, P00.7, O69.4XX3, P84, O86.13, O64.9, P60, P36.19, O60.20X0, P02.60, O60, O64.5XX0, P01.0, O64.2XX1, P07.37, P74.421, P00.8, P22.9, P77.9, P74.21, O60.12, P02.9, O64.4XX3, O62.9, P07.00, O65.5, Z05.89, O64.8XX0, O69.1XX1, Z38, P03.0, P04.41, O60.23X0, O69.5XX0, Z37.4, O66.1, O64.5XX1, Z39.0, P12.4, P91.8, P74.41 |

**Supplemental Table 3. ICD-9 and ICD-10 codes are used to classify vaginal vs. cesarean deliveries**

Delivery type (vaginal vs cesarean) was classified using International Classification of Diseases, Ninth Revision (ICD-9) and Tenth Revision (ICD-10) diagnosis and procedure codes. When delivery codes were unavailable, deliveries were assumed to be vaginal to avoid misclassification, consistent with prior literature and reimbursement incentives for cesarean coding.^1^

|  | ICD-9 | ICD-10 |
| --- | --- | --- |
| Vaginal Birth | V39.2, V32.2, V31.00, V36.00, V36.2, V34.00, V30.2, V39.00,650,V37.00, V33.2, V33.00, V31.2, V34.2,  V36.01,665.4, V30.00, V32.00, V35.00 | Z38.68, Z38.7, Z38.61, Z38.30, O80, Z38.69, Z38.4, O71.4, Z38.1, Z38.00, O83, O81 |
| Cesarean Birth | V31.01, 649.8, V37.01, 669.7, V33.01, V35.01, V30.01, 763.4, 649.82, 669.7, 649.81, V39.01, V32.01, 669.71, V34.01 | O75.82, Z38.62, Z38.01, O90.0, Z38.69, Z38.31, P03.4, O82 |

**Supplemental Table 4. List of opioid analgesics included in the final analytic cohort**

The list of opioid analgesics was compiled from IQVIA PharMetrics® Plus for Academics Closed Health Plan claims database. MME conversion factors were obtained from the 2022 CDC Clinical Practice Guideline for Prescribing Opioids for Pain.^2^ Final selections were reviewed and validated by clinical experts, including obstetrician-gynecologists, to ensure accuracy and clinical relevance for the study population.

| Generic Name |
| --- |
| OXYCODONE W/ ACETAMINOPHEN |
| HYDROCODONE-ACETAMINOPHEN |
| ACETAMINOPHEN W/ CODEINE |
| OXYCODONE HCL |
| HYDROMORPHONE HCL |
| TRAMADOL HCL |
| MORPHINE SULFATE |
| HYDROCODONE-IBUPROFEN |
| OXYCODONE-ASPIRIN |
| CODEINE SULFATE |
| TRAMADOL-ACETAMINOPHEN |

**Supplemental Figure 1. Schematic of the study design**

To define the analytic cohort, we required female patients to have continuous enrollment and complete medical, and pharmacy claims for at least 90 days prior to delivery and 42 days following delivery. All eligible pregnancy episodes were included, regardless of previous opioid exposure or postpartum opioid dispensing.

Pregnancy

-90 days

**Exposure: Postpartum opioid prescription**

≥ 42 days postpartum (post)

Delivery

**Supplemental Figure 2. Flowchart depicting inclusion and exclusion criteria for final analytic cohort**

Patients aged 12 to 59 years (**n=5,036,338**)

Number of US enrollees in the study sample (January 2006 - December 2023): IQVIA database (**n=7,657,187)**

Number of pregnancies recorded with delivery type information, such as cesarean or vaginal **(n=361,165)**

Pregnancy episodes without information from the 90 days prior to delivery and 42 days after were excluded, n= 381,399

Number of pregnancies that resulted in a **cesarean delivery (n=35,918)**

Number of pregnancies that resulted in **vaginal delivery (n=325,247)**

Number of pregnancy episodes with historical data prior to and after delivery (**n=521,372**)

Female patients (n=785,099) with information regarding pregnancy and delivery for **(n=902,771) pregnancy episodes**

Patients aged below 12 or above 59 were excluded, n=2,620,849

Female patients aged 12 to 59 years (**n=4,891,514**)

We lose a total of 4,106,415 female patients because of missing pregnancy and/or delivery codes, or because their deliveries occurred outside the 2006–2015 study period.

Pregnancy episodes were excluded (n=160,207) based on the following criteria: invalid days' supply values (0 or >999), non-oral opioid formulations, episodes with >90 MME/day, missing state identifier, and states that implemented PDMPs prior to or during 2006.

Non-female patients were excluded, n= 144,824

Instead of concentrating on individual patients, we focused on pregnancy episodes from 2006 to 2015, noting that female patients can experience multiple pregnancies during their lifetime.

**Supplemental Figure 3.** **Schematic of the multiple pregnancy logic**

To ensure pregnancies were distinct episodes, we required at least 90 days between the first recorded pregnancy diagnosis and the associated delivery, as well as a minimum of 210 days between consecutive deliveries. This time frame accounts for a 6-week postpartum period, in addition to a minimum of 24 weeks of gestation required for fetal viability .^3,4^ We only included pregnancy episodes that had recorded pregnancy and delivery dates.

-90 days

1^st^ Delivery

2^nd^ Pregnancy Diagnosis

≥ 210 days

2^nd^ Delivery

≥ 42 days postpartum (post)

**Exposure: Postpartum opioid prescription**

**Supplementary Table 5: ICD-9 and ICD-10 diagnosis codes used to identify OUD**

Opioid use disorder (OUD) was identified using ICD-9 and ICD-10 diagnosis codes recorded in medical claims during the 90 days prior to delivery. Code selection was guided by previously published literature and refined through input from a clinical expert and a practicing obstetrician–gynecologist.^5^

| **ICD-9** | **ICD-10** |
| --- | --- |
| 304.0x, 305.5x, 648.3x | F11.x, T40.x |

**Supplemental Table 6. Year of Prescription Drug Monitoring Program (PDMP) implementation by state**

| Year of Implementation | States |
| --- | --- |
| 1982^a^ | HI |
| 2004^a^ | NV, WV, WY |
| 2005^a^ | KY, ME, NM |
| 2006^a^ | OH, OK, TN, UT, VA |
| 2007 | AL, ND |
| 2008 | AZ, CO, CT, ID, IL, IN, NC, SC |
| 2009 | CA, IA, LA, VT |
| 2010 | MA, MN |
| 2011 | FL, KS, MI, MS, NE, OR |
| 2012 | AK, DE, MT, NJ, RI, SD, TX, WA |
| 2013 | AR, GA, NY, WI |
| 2014 | MD, NH |
| 2016 | DC, PA |
| 2017 | MO |

This table lists the year in which each state implemented its PDMP. States with PDMP implementation before or during 2006 were excluded from the primary analysis. Information on PDMP enactment years was obtained from.^6^

^a^ States with PDMP implementation before and during 2006 were excluded from the primary analysis.

**Supplementary Methods:** Difference-in-Differences Specification

Cohort construction, enrollment criteria, postpartum outcome definitions, exclusion rules and risk stratification are described in the main methods section. This supplement provides additional clarification regarding the analytic time structure and the difference-in-difference (DiD) implementation.

The unit of analysis was pregnancy episode. Each pregnancy episode was assigned to single analytic year based on delivery year. Postpartum opioids outcomes were measured within 0-42 days of following delivery and attributed to delivery year, even when claims extended to subsequent year of PDMP establishment. Pregnancies delivered in the implementation year or later were considered exposed, while pregnancies from earlies years served has the controls. States that had not yet implemented PDMP each year served as the comparison group. The analytic sample included deliveries from 2006 through 2015.

Group time average treatment effects were estimated using Callaway and Sant’Anna doubly robust estimator for repeated cross-sectional data. Standard errors were clustered at the state level. The cohort-specific treatment effect is defined as:

$\hat{ATT}\left( g,t \right)=E\left[ Y_{it}\left( g \right)-Y_{it}\left( o \right) \right|G_{i}=g]$,

Where $Y_{it}$ denotes the postpartum opioid outcome for pregnancy I in year t, $G_{i}$ denotes the PDMP adoption year (treatment cohort), and g indexes the cohort first treated in year g.

Dynamic treatment effects were estimated over an event time window of -2 to +3 years relative PDMP implementation to assess the pre-treatment trends and post-implementation effects. Replication code and analytic scripts are publicly available at this GitHub link: <https://github.com/Trilochan-Tadi/Impact-of-Prescription-Drug-Monitoring-Programs-on-Postpartum-Opioid-Prescribing-Practices-Code.git>

**Supplementary Table 7: ICD-9 and ICD-10 Diagnosis Codes Used to Identify Medical and Mental Health Comorbidities**

| **Covariate** | **ICD-9** | **ICD-10** |
| --- | --- | --- |
| Hypertension | 401–405, 642.x | I10–I15, O10, O11, O13 |
| Asthma | 493.xx | J45.x, J46 |
| Diabetes | 250.xx, 648.0x, 648.8x | E10.x, E11.x, O24.0–O24.1, O24.4 |
| Depression | 296.2x–296.3x, 296.5x, 300.4, 309.x, 311 | F31.x, F32.x, F33.x, F34.1, F43.2x |

Comorbidities were identified using ICD-9 and ICD-10 diagnosis codes appearing in medical claims within the 90-day period prior to delivery. Code selections were informed by prior literature and refined in consultation with a clinical expert and a practicing obstetrician–gynecologist.^5,7^

**Supplementary Table 8: Full Covariate-Adjusted Difference-in-Differences Estimates for Low-Risk Pregnancy Episodes**

| Year pre/post law implementation | ATT^a^ | 95% CI |
| --- | --- | --- |
|  |  |  |
| Total MME^b^ per day |  |  |
| -2 | -0.2009 | -2.1016 to 1.6999 |
| -1 | 0.6537 | -1.6311 to 2.9386 |
| 0 | -0.3504 | -3.268 to 2.5673 |
| 1 | 0.6956 | -2.1442 to 3.5355 |
| 2 | 1.3275 | -1.317 to 3.972 |
| 3 | 0.7268 | -3.0583 to 4.5119 |
| Total pill dispensation |  |  |
| -2 | -0.0169 | -0.9418 to 0.908 |
| -1 | 0.2122 | -0.5335 to 0.958 |
| 0 | -0.1989 | -1.7913 to 1.3935 |
| 1 | 0.2254 | -1.2614 to 1.7121 |
| 2 | 0.4726 | -1.0562 to 2.0013 |
| 3 | 0.2313 | -1.6014 to 2.064 |
| Average days’ supply |  |  |
| -2 | -0.0027 | -0.1191 to 0.1138 |
| -1 | 0.0204 | -0.0757 to 0.1165 |
| 0 | -0.0251 | -0.218 to 0.1678 |
| 1 | 0.0189 | -0.2132 to 0.2511 |
| 2 | 0.0431 | -0.2284 to 0.3147 |
| 3 | 0.0113 | -0.2716 to 0.2943 |
| Total number of prescriptions filled |  |  |
| -2 | -0.0028 | -0.0345 to 0.0288 |
| -1 | 0.0081 | -0.0198 to 0.0359 |
| 0 | -0.0067 | -0.0617 to 0.0483 |
| 1 | 0.0078 | -0.0481 to 0.0637 |
| 2 | 0.0155 | -0.0488 to 0.0799 |
| 3 | 0.0066 | -0.0605 to 0.073 |

This table presents the complete set of estimates from the adjusted model; in the main manuscript, we highlight event times −1, 0, and +1 around PDMP implementation. Values represent covariate adjusted DID estimates using the Callaway and Sant’Anna doubly robust estimator. Models adjust for age at delivery and medical and mental health comorbidities, including asthma, hypertension, diabetes, and depression. Estimates are shown for event times −2 through 3 relatives to PDMP implementation. All outcomes were measured within the 42-day postpartum period (0–42 days following delivery) and aggregated at the pregnancy-episode level.

Abbreviations: CI, confidence interval; DID, difference-in-differences; PDMP, prescription drug monitoring program.

^a^ ATT: average treatment effect
^b^ MME: morphine milligram equivalents

| Year pre/post law implementation | ATT^a^ | 95% CI |
| --- | --- | --- |
|  |  |  |
| Total MME^b^ per day |  |  |
| -2 | 1.2136 | -14.9481 to 17.3752 |
| -1 | -2.0926 | -12.1732 to 7.9881 |
| 0 | 1.1138 | -7.4562 to 9.6838 |
| 1 | 1.6612 | -15.5896 to 18.912 |
| 2 | 1.76 | -13.1305 to 16.6505 |
| 3 | -2.7907 | -19.5393 to 13.9578 |
| Total pill dispensation |  |  |
| -2 | 2.3647 | -7.5141 to 12.2436 |
| -1 | -2.7074 | -10.8876 to 5.4729 |
| 0 | 0.7094 | -6.8424 to 8.2611 |
| 1 | 0.5334 | -20.5983 to 21.6651 |
| 2 | -0.4922 | -10.919 to 9.9346 |
| 3 | -1.547 | -12.6347 to 9.5407 |
| Average days’ supply |  |  |
| -2 | 0.6277 | -1.509 to 2.7643 |
| -1 | -0.5469 | -2.517 to 1.4232 |
| 0 | 0.2579 | -1.8165 to 2.3322 |
| 1 | 0.3324 | -3.8879 to 4.5528 |
| 2 | 0.0714 | -2.1551 to 2.2979 |
| 3 | 0.1842 | -2.9312 to 3.2996 |
| Total number of prescriptions filled |  |  |
| -2 | 0.0752 | -0.255 to 0.4054 |
| -1 | -0.0701 | -0.2955 to 0.1554 |
| 0 | 0.0342 | -0.2549 to 0.3234 |
| 1 | 0.0219 | -0.3906 to 0.4345 |
| 2 | 0.0374 | -0.3036 to 0.3783 |
| 3 | -0.0451 | -0.3601 to 0.2699 |

**Supplementary Table 9: Full Covariate-Adjusted Difference-in-Differences Estimates for High-Risk Pregnancy Episodes**

This table presents the complete set of estimates from the adjusted model; in the main manuscript, we highlight event times −1, 0, and +1 around PDMP implementation. Values represent covariate adjusted DID estimates using the Callaway and Sant’Anna doubly robust estimator. Models adjust for age at delivery and medical and mental health comorbidities, including asthma, hypertension, diabetes, and depression. Estimates are shown for event times −2 through 3 relative to PDMP implementation. All outcomes were measured within the 42-day postpartum period (0–42 days following delivery) and aggregated at the pregnancy-episode level.

Abbreviations: CI, confidence interval; DID, difference-in-differences; PDMP, prescription drug monitoring program.

^a^ ATT: average treatment effect
^b^ MME: morphine milligram equivalents

**Supplementary Table 10: Full Unadjusted Difference-in-Differences Estimates for Low-Risk Pregnancy Episodes**

| Year pre/post law implementation | ATT^a^ | 95% CI |
| --- | --- | --- |
|  |  |  |
| Total MME^b^ per day |  |  |
| -2 | -0.1949 | -2.1808 to 1.791 |
| -1 | 0.4139 | -1.4852 to 2.3129 |
| 0 | -0.3395 | -3.2207 to 2.5417 |
| 1 | 0.3453 | -2.3284 to 3.0189 |
| 2 | 0.5956 | -2.4888 to 3.6801 |
| 3 | 0.0309 | -3.611 to 3.6728 |
| Total pill dispensation |  |  |
| -2 | -0.0675 | -1.2019 to 1.0668 |
| -1 | 0.1627 | -0.7244 to 1.0499 |
| 0 | -0.2112 | -1.9203 to 1.4978 |
| 1 | 0.1005 | -1.4506 to 1.6516 |
| 2 | 0.2503 | -1.703 to 2.2036 |
| 3 | -0.0218 | -2.0021 to 1.9585 |
| Average days’ supply |  |  |
| -2 | -0.0079 | -0.1404 to 0.1246 |
| -1 | 0.0154 | -0.0952 to 0.1259 |
| 0 | -0.0239 | -0.2454 to 0.1976 |
| 1 | 0.012 | -0.2109 to 0.2349 |
| 2 | 0.0317 | -0.2503 to 0.3136 |
| 3 | -0.0039 | -0.2837 to 0.2759 |
| Total number of prescriptions filled |  |  |
| -2 | -0.0033 | -0.0398 to 0.0332 |
| -1 | 0.0058 | -0.0295 to 0.0411 |
| 0 | -0.0062 | -0.0643 to 0.0519 |
| 1 | 0.0049 | -0.0537 to 0.0635 |
| 2 | 0.0096 | -0.0666 to 0.0858 |
| 3 | 0.0006 | -0.0701 to 0.0714 |

This table presents unadjusted estimates from the difference-in-differences models evaluating the association between PDMP implementation and postpartum opioid prescribing. Outcomes include total MME per day per delivery, total pill dispensation per delivery, average days’ supply per delivery, and total number of prescriptions per delivery. Results are reported as ATT with corresponding 95% CI across event-study years (−2 to +3 relative to implementation). All outcomes were measured within the 42-day postpartum period (0–42 days following delivery) and aggregated at the pregnancy-episode level.

Abbreviations: CI, confidence interval; PDMP, prescription drug monitoring program.

^a^ ATT: average treatment effect
^b^ MME: morphine milligram equivalents

| Year pre/post law implementation | ATT^a^ | 95% CI |
| --- | --- | --- |
|  |  |  |
| Total MME^b^ per day |  |  |
| -2 | -0.2009 | -2.157 to 1.7553 |
| -1 | 0.6537 | -1.4544 to 2.7618 |
| 0 | -0.3504 | -3.0577 to 2.357 |
| 1 | 0.6956 | -2.0072 to 3.3985 |
| 2 | 1.3275 | -1.2866 to 3.9416 |
| 3 | 0.7268 | -3.0976 to 4.5512 |
| Total pill dispensation |  |  |
| -2 | -0.0169 | -0.9724 to 0.9386 |
| -1 | 0.2122 | -0.5928 to 1.0173 |
| 0 | -0.1989 | -1.9854 to 1.5875 |
| 1 | 0.2254 | -1.4367 to 1.8874 |
| 2 | 0.4726 | -1.1725 to 2.1177 |
| 3 | 0.2313 | -1.6411 to 2.1036 |
| Average days’ supply |  |  |
| -2 | -0.0027 | -0.1163 to 0.111 |
| -1 | 0.0204 | -0.0793 to 0.12 |
| 0 | -0.0251 | -0.242 to 0.1918 |
| 1 | 0.0189 | -0.2 to 0.2379 |
| 2 | 0.0431 | -0.1883 to 0.2746 |
| 3 | 0.0113 | -0.2643 to 0.287 |
| Total number of prescriptions filled |  |  |
| -2 | -0.0028 | -0.0384 to 0.0327 |
| -1 | 0.0081 | -0.0208 to 0.037 |
| 0 | -0.0067 | -0.0616 to 0.0482 |
| 1 | 0.0078 | -0.0433 to 0.0589 |
| 2 | 0.0155 | -0.0429 to 0.0739 |
| 3 | 0.0066 | -0.0667 to 0.08 |

**Supplementary Table 11: Covariate-Adjusted Difference-in-Differences Estimates for Low-Risk Pregnancy Episodes (≥90 MME per Day Claims Model)**

This table presents covariate-adjusted DiD estimates evaluating the association between PDMP implementation and postpartum opioid prescribing outcomes. Outcomes include total MME per day per delivery, total pills dispensed per delivery, average days’ supply per delivery, and total number of prescriptions per delivery. Results are reported as ATT with corresponding 95% CI across event-study years (−2 to +3 relative to implementation). Estimates were obtained using the Callaway and Sant’Anna doubly robust estimator and adjusted for age at delivery and medical and mental health comorbidities, including asthma, hypertension, diabetes, and depression. All outcomes were measured within the 42-day postpartum period (0–42 days following delivery) and aggregated at the pregnancy-episode level.

Abbreviations: CI, confidence interval; DID, difference-in-differences; PDMP, prescription drug monitoring program.

^a^ ATT: average treatment effect

^b^ MME: morphine milligram equivalents

| Year pre/post law implementation | ATT^a^ | 95% CI |
| --- | --- | --- |
|  |  |  |
| Total MME^b^ per day |  |  |
| -2 | -0.2183 | -1.966 to 1.5293 |
| -1 | 0.8272 | -0.8522 to 2.5066 |
| 0 | -0.3637 | -3.5107 to 2.7832 |
| 1 | 0.3455 | -2.2684 to 2.9595 |
| 2 | 0.6283 | -2.0761 to 3.3328 |
| 3 | 0.2568 | -2.8985 to 3.4121 |
| Total pill dispensation |  |  |
| -2 | -0.0367 | -0.922 to 0.8486 |
| -1 | 0.3138 | -0.3675 to 0.9951 |
| 0 | -0.1948 | -1.9225 to 1.5329 |
| 1 | 0.0664 | -1.2073 to 1.34 |
| 2 | 0.1455 | -1.5991 to 1.8901 |
| 3 | -0.0093 | -1.6747 to 1.6562 |
| Average days’ supply |  |  |
| -2 | -0.0016 | -0.1392 to 0.1359 |
| -1 | 0.03 | -0.0737 to 0.1336 |
| 0 | -0.0233 | -0.2331 to 0.1865 |
| 1 | 0.0011 | -0.2124 to 0.2145 |
| 2 | 0.0092 | -0.2926 to 0.3111 |
| 3 | -0.013 | -0.29 to 0.264 |
| Total number of prescriptions filled |  |  |
| -2 | -0.0022 | -0.0343 to 0.0298 |
| -1 | 0.0107 | -0.0132 to 0.0347 |
| 0 | -0.0063 | -0.0627 to 0.0501 |
| 1 | 0.0037 | -0.0489 to 0.0562 |
| 2 | 0.0065 | -0.0611 to 0.074 |
| 3 | 0.0002 | -0.0634 to 0.0639 |

**Supplementary Table 12: Covariate-Adjusted Subgroup Difference-in-Differences Estimates for Vaginal Deliveries in Low-Risk Pregnancy Episodes.**

This table reports results from subgroup analyses restricted to patients who had a vaginal delivery. Outcomes include total MME per day per delivery, total pill dispensation per delivery, average days’ supply per delivery, and total number of prescriptions per delivery. Results are reported as ATT with corresponding 95% CI across event-study years (−2 to +3 relative to implementation). Values represent covariate adjusted DID estimates using the Callaway and Sant’Anna doubly robust estimator. Models adjust for age at delivery and medical and mental health comorbidities, including asthma, hypertension, diabetes, and depression. All outcomes were measured within the 42-day postpartum period (0–42 days following delivery) and aggregated at the pregnancy-episode level.

Abbreviations: CI, confidence interval; DID, difference-in-differences; PDMP, prescription drug monitoring program.

^a^ ATT: average treatment effect
^b^ MME: morphine milligram equivalents

**Supplementary Table 13: Covariate-Adjusted Subgroup Difference-in-Differences Estimates for Cesarean Deliveries in Low-Risk Pregnancy Episodes.**

| Year pre/post law implementation | ATT^a^ | 95% CI |
| --- | --- | --- |
|  |  |  |
| Total MME^b^ per day |  |  |
| -2 | 1.5533 | -10.3619 to 13.4685 |
| -1 | -0.757 | -12.5791 to 11.0653 |
| 0 | -0.239 | -21.184 to 20.7066 |
| 1 | 1.8305 | -10.2757 to 13.9367 |
| 2 | 3.7653 | -10.2259 to 17.7566 |
| 3 | 1.1418 | -19.1536 to 21.4372 |
| Total pill dispensation |  |  |
| -2 | 0.9427 | -4.0312 to 5.9167 |
| -1 | -0.632 | -6.865 to 5.6015 |
| 0 | -0.145 | -9.3219 to 9.0317 |
| 1 | 0.6732 | -6.2262 to 7.5726 |
| 2 | 1.4439 | -5.6518 to 8.5396 |
| 3 | 0.4013 | -9.8748 to 10.6774 |
| Average days’ supply |  |  |
| -2 | 0.0806 | -0.6067 to 0.768 |
| -1 | -0.053 | -1.1604 to 1.0549 |
| 0 | -0.023 | -1.0396 to 0.9936 |
| 1 | 0.0763 | -0.886 to 1.0386 |
| 2 | 0.1262 | -1.0052 to 1.2576 |
| 3 | -0.009 | -1.5962 to 1.579 |
| Total number of prescriptions filled |  |  |
| -2 | 0.0137 | -0.1317 to 0.159 |
| -1 | -0.012 | -0.2233 to 0.2002 |
| 0 | -0.008 | -0.3868 to 0.3706 |
| 1 | 0.0165 | -0.1908 to 0.2239 |
| 2 | 0.0397 | -0.1929 to 0.2724 |
| 3 | 0.0038 | -0.3181 to 0.3258 |

This table reports results from subgroup analyses restricted to patients who had caesarean delivery. Outcomes include total MME per day per delivery, total pill dispensation per delivery, average days’ supply per delivery, and total number of prescriptions per delivery. Results are reported as ATT with corresponding 95% CI across event-study years (−2 to +3 relative to implementation). Values represent covariate adjusted DID estimates using the Callaway and Sant’Anna doubly robust estimator. Models adjust for age at delivery and medical and mental health comorbidities, including asthma, hypertension, diabetes, and depression. All outcomes were measured within the 42-day postpartum period (0–42 days following delivery) and aggregated at the pregnancy-episode level.

Abbreviations: CI, confidence interval; DID, difference-in-differences; PDMP, prescription drug monitoring program.

^a^ ATT: average treatment effect
^b^ MME: morphine milligram equivalents

**Supplementary Table 14: Full Unadjusted Difference-in-Differences Estimates for High-Risk Pregnancy Episodes**

| Year pre/post law implementation | ATT^a^ | 95% CI |
| --- | --- | --- |
|  |  |  |
| Total MME^b^ per day |  |  |
| -2 | 0.9882 | -16.8836 to 18.8599 |
| -1 | -1.9022 | -10.4658 to 6.6615 |
| 0 | 0.5835 | -8.083 to 9.25 |
| 1 | 1.3322 | -16.6361 to 19.3005 |
| 2 | 1.6355 | -12.5198 to 15.7909 |
| 3 | -2.9517 | -19.1614 to 13.2579 |
| Total pill dispensation |  |  |
| -2 | 1.9996 | -7.3932 to 11.3924 |
| -1 | -2.487 | -10.5793 to 5.6052 |
| 0 | 0.2087 | -7.9377 to 8.3551 |
| 1 | 0.0284 | -19.587 to 19.6439 |
| 2 | -0.737 | -10.4706 to 8.9965 |
| 3 | -1.7067 | -11.6032 to 8.1898 |
| Average days’ supply |  |  |
| -2 | 0.5691 | -1.8549 to 2.9931 |
| -1 | -0.5577 | -2.7921 to 1.6768 |
| 0 | 0.2425 | -1.8688 to 2.3537 |
| 1 | 0.3021 | -3.9918 to 4.596 |
| 2 | 0.0749 | -2.577 to 2.7268 |
| 3 | 0.2156 | -3.3185 to 3.7497 |
| Total number of prescriptions filled |  |  |
| -2 | 0.0722 | -0.292 to 0.4364 |
| -1 | -0.0689 | -0.2884 to 0.1506 |
| 0 | 0.0253 | -0.2736 to 0.3243 |
| 1 | 0.0219 | -0.4071 to 0.4508 |
| 2 | 0.0412 | -0.2695 to 0.3519 |
| 3 | -0.0393 | -0.3561 to 0.2775 |

This table presents unadjusted estimates from the difference-in-differences models evaluating the association between PDMP implementation and postpartum opioid prescribing. Outcomes include total MME per day per delivery, total pill dispensation per delivery, average days’ supply per delivery, and total number of prescriptions per delivery. Results are reported as ATT with corresponding 95% CI across event-study years (−2 to +3 relative to implementation). All outcomes were measured within the 42-day postpartum period (0–42 days following delivery) and aggregated at the pregnancy-episode level.

Abbreviations: CI, confidence interval; PDMP, prescription drug monitoring program.

^a^ ATT: average treatment effect
^b^ MME: morphine milligram equivalents

| Year pre/post law implementation | ATT^a^ | 95% CI lower bound |
| --- | --- | --- |
|  |  |  |
| Total MME^b^ per day |  |  |
| -2 | 5.34 | -73.626 to 84.308 |
| -1 | -2.2 | -33.652 to 29.202 |
| 0 | 2.5 | -17.747 to 22.742 |
| 1 | 5.36 | -20.282 to 30.927 |
| 2 | 9.37 | -26.766 to 45.506 |
| 3 | -1.6 | -37.685 to 34.559 |
| Total pill dispensation |  |  |
| -2 | 6.17 | -23.256 to 35.592 |
| -1 | -3.1 | -22.762 to 16.616 |
| 0 | 0.02 | -14.558 to 14.592 |
| 1 | 0.91 | -15.993 to 17.805 |
| 2 | 1.29 | -15.419 to 17.989 |
| 3 | -1.7 | -23.001 to 19.664 |
| Average days’ supply |  |  |
| -2 | 1.3 | -4.277 to 6.883 |
| -1 | -0.5 | -4.578 to 3.521 |
| 0 | 0.03 | -4.908 to 4.958 |
| 1 | 0.4 | -3.619 to 4.427 |
| 2 | 0.29 | -3.907 to 4.477 |
| 3 | 0.38 | -5.765 to 6.528 |
| Total number of prescriptions filled |  |  |
| -2 | 0.17 | -0.861 to 1.196 |
| -1 | -0.1 | -0.506 to 0.406 |
| 0 | 0.04 | -0.491 to 0.568 |
| 1 | 0.06 | -0.411 to 0.527 |
| 2 | 0.13 | -0.386 to 0.644 |
| 3 | -0 | -0.667 to 0.607 |

**Supplementary Table 15 : Full Difference-in-Differences Estimates for High-Risk Pregnancy Episodes (≥90 MME per Day Claims Model)**

This table presents unadjusted estimates from the difference-in-differences models evaluating the association between PDMP implementation and postpartum opioid prescribing. Outcomes include total MME per day per delivery, total pill dispensation per delivery, average days’ supply per delivery, and total number of prescriptions per delivery. Results are reported as ATT with corresponding 95% CI across event-study years (−2 to +3 relative to implementation). Values represent covariate-adjusted DID estimates using the Callaway and Sant’Anna doubly robust estimator. Models adjust for age at delivery and medical and mental health comorbidities, including asthma, hypertension, diabetes, and depression. All outcomes were measured within the 42-day postpartum period (0–42 days following delivery) and aggregated at the pregnancy-episode level.

Abbreviations: CI, confidence interval; DID, difference-in-differences; PDMP, prescription drug monitoring program.

^a^ ATT: average treatment effect
^b^ MME: morphine milligram equivalents

| Year pre/post law implementation | ATT^a^ | 95% CI |
| --- | --- | --- |
|  |  |  |
| Total MME^b^ per day |  |  |
| -2 | 2.3051 | -17.1176 to 21.7277 |
| -1 | -1.3044 | -14.3731 to 11.7643 |
| 0 | 0.6345 | -9.5744 to 10.8435 |
| 1 | 0.4824 | -15.5186 to 16.4835 |
| 2 | 2.012 | -11.4454 to 15.4693 |
| 3 | -3.067 | -22.6646 to 16.5305 |
| Total pill dispensation |  |  |
| -2 | 2.404 | -8.6698 to 13.4777 |
| -1 | -1.9624 | -11.4761 to 7.5512 |
| 0 | 0.9814 | -6.2426 to 8.2054 |
| 1 | 0.0812 | -29.8093 to 29.9717 |
| 2 | 0.425 | -9.8561 to 10.7061 |
| 3 | -1.6895 | -13.7466 to 10.3675 |
| Average days’ supply |  |  |
| -2 | 0.4783 | -1.4692 to 2.4258 |
| -1 | -0.4215 | -2.4222 to 1.5792 |
| 0 | 0.2807 | -1.5109 to 2.0724 |
| 1 | 0.2814 | -7.5706 to 8.1333 |
| 2 | 0.326 | -1.7395 to 2.3914 |
| 3 | 0.2355 | -2.0804 to 2.5514 |
| Total number of prescriptions filled |  |  |
| -2 | 0.0869 | -0.2996 to 0.4734 |
| -1 | -0.0542 | -0.3348 to 0.2263 |
| 0 | 0.0293 | -0.2168 to 0.2754 |
| 1 | 0.0069 | -0.4948 to 0.5086 |
| 2 | 0.048 | -0.3024 to 0.3985 |
| 3 | -0.0528 | -0.4758 to 0.3703 |

**Supplementary Table 16: Covariate-Adjusted Subgroup Difference-in-Differences Estimates for Vaginal Deliveries in High-Risk Pregnancy Episodes**

This table reports results from subgroup analyses restricted to patients who had a vaginal delivery. Outcomes include total MME per day per delivery, total pill dispensation per delivery, average days’ supply per delivery, and total number of prescriptions per delivery. Results are reported as ATT with corresponding 95% CI across event-study years (−2 to +3 relative to implementation). Values represent covariate adjusted DID estimates using the Callaway and Sant’Anna doubly robust estimator. Models adjust for age at delivery and medical and mental health comorbidities, including asthma, hypertension, diabetes, and depression. All outcomes were measured within the 42-day postpartum period (0–42 days following delivery) and aggregated at the pregnancy-episode level.

Abbreviations: CI, confidence interval; DID, difference-in-differences; PDMP, prescription drug monitoring program.

^a^ ATT: average treatment effect
^b^ MME: morphine milligram equivalents

| Year pre/post law implementation | ATT^a^ | 95% CI lower bound |
| --- | --- | --- |
|  |  |  |
| Total MME^b^ per day |  |  |
| -2 | -1.6896 | -21.2231 to 17.8439 |
| -1 | -4.5511 | -24.4862 to 15.384 |
| 0 | 2.4958 | -112.2701 to 117.2618 |
| 1 | 4.9526 | -25.5846 to 35.4898 |
| 2 | 1.5562 | -32.7009 to 35.8133 |
| 3 | -0.2352 | -55.548 to 55.0775 |
| Total pill dispensation |  |  |
| -2 | 2.4435 | -11.0332 to 15.9202 |
| -1 | -4.6815 | -20.2628 to 10.8998 |
| 0 | -1.0204 | -89.214 to 87.1732 |
| 1 | 0.9035 | -11.4266 to 13.2337 |
| 2 | -3.5891 | -18.6096 to 11.4314 |
| 3 | 0.3171 | -29.2996 to 29.9338 |
| Average days’ supply |  |  |
| -2 | 1.384 | -3.5249 to 6.293 |
| -1 | -1.1703 | -5.8602 to 3.5196 |
| 0 | 0.2748 | -8.9188 to 9.4685 |
| 1 | 0.3766 | -4.43 to 5.1831 |
| 2 | -0.6225 | -7.7967 to 6.5517 |
| 3 | 0.5148 | -7.4858 to 8.5154 |
| Total number of prescriptions filled |  |  |
| -2 | 0.0136 | -0.1388 to 0.1661 |
| -1 | -0.0121 | -0.2389 to 0.2147 |
| 0 | -0.0071 | -0.3532 to 0.3389 |
| 1 | 0.013 | -0.1957 to 0.2217 |
| 2 | 0.0405 | -0.2287 to 0.3097 |
| 3 | 0.0054 | -0.314 to 0.3248 |

**Supplementary Table 17: Covariate-Adjusted Subgroup Difference-in-Differences Estimates for Cesarean Deliveries in High-Risk Pregnancy Episodes**

This table reports results from subgroup analyses restricted to patients who had caesarean delivery. Outcomes include total morphine milligram equivalents per day per delivery, total pill dispensation per delivery, average days’ supply per delivery, and total number of prescriptions per delivery. Results are reported as ATT with corresponding 95% CI across event-study years (−2 to +3 relative to implementation). Values represent covariate adjusted DID estimates using the Callaway and Sant’Anna doubly robust estimator. Models adjust for age at delivery and medical and mental health comorbidities, including asthma, hypertension, diabetes, and depression. All outcomes were measured within the 42-day postpartum period (0–42 days following delivery) and aggregated at the pregnancy-episode level.

Abbreviations: CI, confidence interval; DID, difference-in-differences; PDMP, prescription drug monitoring program.

^a^ ATT: average treatment effect
^b^ MME: morphine milligram equivalents

**Supplementary References**

1. The Web’s Free 2025 ICD-10-CM/PCS Medical Coding Reference. Accessed September 6, 2025. https://www.icd10data.com/

2. Dowell D. CDC Clinical Practice Guideline for Prescribing Opioids for Pain — United States, 2022. *MMWR Recomm Rep*. 2022;71. doi:10.15585/mmwr.rr7103a1

3. ACOG Committee Opinion No. 736: Optimizing Postpartum Care. *Obstet Gynecol*. 2018;131(5):e140-e150. doi:10.1097/AOG.0000000000002633

4. American College of Obstetricians and Gynecologists, Society for Maternal-Fetal Medicine. Obstetric Care consensus No. 6: Periviable Birth. *Obstet Gynecol*. 2017;130(4):e187-e199. doi:10.1097/AOG.0000000000002352

5. Sun EC, Darnall BD, Baker LC, Mackey S. Incidence of and Risk Factors for Chronic Opioid Use Among Opioid-Naive Patients in the Postoperative Period. *JAMA Intern Med*. 2016;176(9):1286-1293. doi:10.1001/jamainternmed.2016.3298

6. Gihleb R, Giuntella O, Zhang N. The Effect of Mandatory Access Prescription Drug Monitoring Programs on Foster Care Admissions. *J Hum Resour*. Published online December 6, 2019. doi:10.3368/jhr.57.1.0918-9729R2

7. Bateman BT, Mhyre JM, Hernandez-Diaz S, et al. Development of a Comorbidity Index for Use in Obstetric Patients. *Obstet Gynecol*. 2013;122(5):10.1097/AOG.0b013e3182a603bb. doi:10.1097/AOG.0b013e3182a603bb
